# CircMYO10 promotes osteosarcoma progression by regulating miR-370-3p/RUVBL1 axis to enhance the transcriptional activity of β-catenin/LEF1 complex via effects on chromatin remodeling

**DOI:** 10.1186/s12943-019-1076-1

**Published:** 2019-10-29

**Authors:** Junxin Chen, Gang Liu, Yizheng Wu, Jianjun Ma, Hongfei Wu, Ziang Xie, Shuai Chen, Yute Yang, Shengyu Wang, Panyang Shen, Yifan Fang, Shunwu Fan, Shuying Shen, Xiangqian Fang

**Affiliations:** 1 Department of Orthopaedic Surgery, Sir Run Run Shaw Hospital, Medical College of Zhejiang University & Key Laboratory of Musculoskeletal System Degeneration and Regeneration Translational Research of Zhejiang Province, 3 East Qingchun Road, Hangzhou, 310016 Zhejiang Province China; 2 Department of Spinal Surgery, Orthopaedic Medical Center, Hospital of Zhejiang Armed Police Corps, Jiaxing, Zhejiang Province China; 3 Hangzhou Foreign Language School, Hangzhou, Zhejiang Province China

**Keywords:** Osteosarcoma, Circular RNA, circMYO10, RUVBL1, Wnt/β-catenin signaling, EMT, miR-370-3p, Chromatin remodel, Histone H4K16 acetylation

## Abstract

**Background:**

CircMYO10 is a circular RNA generated by back-splicing of gene MYO10 and is upregulated in osteosarcoma cell lines, but its functional role in osteosarcoma is still unknown. This study aimed to clarify the mechanism of circMYO10 in osteosarcoma.

**Methods:**

CircMYO10 expression in 10 paired osteosarcoma and chondroma tissues was assessed by quantitative reverse transcription polymerase chain reaction (PCR). The function of circMYO10/miR-370-3p/RUVBL1 axis was assessed regarding two key characteristics: proliferation and endothelial–mesenchymal transition (EMT). Bioinformatics analysis, western blotting, real-time PCR, fluorescence in situ hybridization, immunoprecipitation, RNA pull-down assays, luciferase reporter assays, chromatin immunoprecipitation, and rescue experiments were used to evaluate the mechanism. Stably transfected MG63 cells were injected via tail vein or subcutaneously into nude mice to assess the role of circMYO10 in vivo.

**Results:**

CircMYO10 was significantly upregulated, while miR-370-3p was downregulated, in osteosarcoma cell lines and human osteosarcoma samples. Silencing circMYO10 inhibited cell proliferation and EMT in vivo and in vitro. Mechanistic investigations revealed that miR-370-3p targets RUVBL1 directly, and inhibits the interaction between RUVBL1 and β-catenin/LEF1 complex while circMYO10 showed a contrary effect via the inhibition of miR-370-3p. RUVBL1 was found to be complexed with chromatin remodeling and histone-modifying factor TIP60, and lymphoid enhancer factor-1 (LEF1) to promote histone H4K16 acetylation (H4K16Ac) in the vicinity of the promoter region of gene C-myc. Chromatin immunoprecipitation methods showed that miR-370-3p sponge promotes H4K16Ac in the indicated region, which is partially abrogated by RUVBL1 small hairpin RNA (shRNA) while circMYO10 showed a contrary result via the inhibition of miR-370-3p. Either miR-370-3p sponge or ShRUVBL1 attenuated circMYO10-induced phenotypes in osteosarcoma cell lines. MiR-370-3p inhibition abrogated the inhibition of proliferation, EMT of osteosarcoma cells in vitro and in vivo seen upon circMYO10 suppression via Wnt/β-catenin signaling.

**Conclusions:**

CircMYO10 promotes osteosarcoma progression by regulating miR-370-3p/RUVBL1 axis to promote chromatin remodeling and thus enhances the transcriptional activity of β-catenin/LEF1 complex, which indicates that circMYO10 may be a potential therapeutic target for osteosarcoma treatment.

## Background

Osteosarcoma (OS) originates from mesenchymal stem cells. It is the most common malignant bone tumor, mainly occurring during childhood and adolescence [1, 2]. OS features a highly aggressive phenotype, with 75% of OS cells invading nearby tissues [2, 3]. Although the overall 5-year survival rate of patients with OS has increased from 10 to 70% with the application of chemotherapy and surgery over the last 30 years, prognosis remains poor for cases displaying metastasis or drug resistance [4]. Thus, there is an urgent need to find new treatment strategies for OS.

Non-coding RNAs, including microRNAs (miRNAs), long non-coding RNAs (lncRNAs), and circular RNAs (circRNAs), play crucial roles in cell development and disease [5–9]. CircRNA is a class of single-stranded ncRNA derived from exons, introns, or intergenic regions of genes by back-splicing [10, 11]. CircRNA are characterized by a closed loop structure with covalent links between the 5′ and 3′ ends of the RNA. In contrast to linear RNA, circRNA is not sensitive to RNase R [10–14]. CircRNA expression has been widely reported in various species and cell lines [15–19]. Importantly, there is evidence for circRNA involvement in various diseases [20–23]. CircRNA possess miRNA-binding sites and have commonly been reported to act as “microRNA sponges” to modulate gene expression [16, 20, 22]. Emerging evidence indicates that circRNA modulates OS progression [24–27]. However, further research is needed to elucidate the mechanism.

The Wnt/β-catenin signaling pathway is reported to play a crucial role in cell fate specification, proliferation, and migration [28–31]. Dysregulation of Wnt/β-catenin signaling has been implicated in many human cancers including osteosarcoma [32, 33]. β-catenin plays a central role in canonical Wnt signaling and when Wnt binds to a Frizzled family receptor, β-catenin will accumulate in cytoplasm which will result in the nuclear translocation of β-catenin where it acts as a transcriptional coactivator for the TCF/LEF family of transcription factors promoting the expression of Wnt-target genes such as C-myc and CyclinD1 [28–31, 34, 35]. In the absence of Wnt signaling, cytoplasmic β-catenin undergoes ubiquitination, targeting it for proteasomal degradation, which is mediated by a destruction complex composed of Axin, adenomatosis polyposis coli (APC), protein phosphatase 2A (PP2A), glycogen synthase kinase 3 (GSK3), and casein kinase 1α (CK1α) [29, 32, 34, 36].

RUVBL1 (TIP49a, TIP, Pontin, Pontin52) is a highly conserved AAA+ ATPase containing the conserved Walker A and Walker B motifs, which are responsible for ATP binding and hydrolysis [37, 38]. It’s reported that RUVBL1 is a coactivator of TCF/LEF which antagonizes with RUVBL2 in regulation of β-catenin signaling and RUVBL1 binds to almost the same region of β-catenin as RUVBL2 [39, 40]. Moreover, RUVBL1 was frequently shown to be a coactivator for transcriptions of various genes including KAI1, a tumor suppressor gene which inhibits tumor metastasis [41]. While promoting the transcription of tumor suppressors genes, RUVBL1 was revealed to promote β-catenin-mediated neoplastic transformation by forming chromatin remodeling complex with TIP60 and thus promotes histone H4 acetylation in the promoter region of ITF-2 gene and enhances the transcriptional activity of TCF4 [42]. In addition, RUVBL1 is also involved in C-myc-mediated oncogenic transformation [43]. Widely reported as an oncogene, RUVBL1 was shown to promote mutant p53 gain-of-function in osteosarcoma cell line SaoS-2 [43]. All this evidence suggests that RUVBL1 plays an important role in oncogenesis.

In this study, we identified the oncogenic role of circMYO10, a circRNA that is upregulated in OS [44]. Importantly, we reported that miR-370-3p targets RUVBL1 directly and circMYO10 acts as a sponge for miR-370-3p and thus upregulates the expression of RUVBL1 to promote chromatin remodeling at the promoter region of LEF1 target gene enhancing the transcriptional activity of β-catenin/LEF1 complex.

## Methods

### Clinical samples

Samples from 10 chondroma and 10 OS tissues were collected from 20 untreated patients prior to surgery (10 for each group). All patients provided informed written consent authorizing the use of specimens for the intended research. Specimens were identified by the Pathology Department of Sir Run Shaw Hospital according to the criteria established by the World Health Organization. Fresh samples were either pretreated with liquid nitrogen and stored at − 80 °C until RNA extraction, or subjected to formalin fixation and embedded in paraffin for use in procedures such as Fluorescent in Situ Hybridization (FISH).

### Materials

Materials used in this study are described in Additional file 1: Supplementary Materials and Methods.

### Cell culture

The human cell line hFOB1.19 as well as the human OS cell lines 143B, HOS, U2OS, MG-63, and SJSA-1 were purchased from FuHeng Cell Center (Shanghai, China). All cell lines were confirmed to be mycoplasma-free using a Venor GeM Mycoplasma Detection Kit (Minerva Biolabs, Berlin, Germany). All cells were cultured in Dulbecco’s modified Eagle medium (DMEM) supplemented with 10% fetal bovine serum (FBS). All cells were incubated at 5% CO_2_ at either 34 °C (hFOB1.19) or 37 °C (OS cell lines).

### Xenograft tumorigenesis

Four-week-old nude mice were administered subcutaneously with 10^7^ stable MG63 cells suspended in phosphate-buffered saline (PBS) on one side of the lower dorsal flank (*n* = 6 per group). The width and length of the tumor was measured every week for 5 weeks, and tumor volume was calculated according to the formula: volume (mm^3^) = (length × width^2^)/2. Five weeks after injection, mice were sacrificed and tumors were harvested and weighed. About 0.02 g tissues were collected from each tumor for protein extraction and the rest was fixed for further use.

### Tail vein metastasis and bioluminescent imaging

Luminescence-labeled MG63 cells were stably transfected with vector, shcircMYO10, or shcircMYO10 and miR-370-3p sponge. Six-week-old mice were injected with 5 × 10^6^ stable cells via the tail vein (*n* = 5 for each group). Four weeks later, the mice were anesthetized with isoflurane and intraperitoneally injected with 150 mg/kg D-luciferin (Yeason). Fifteen minutes later, tumors expressing luciferase were imaged using an IVIS Spectrum apparatus (Caliper Life Sciences, Hopkinton, MA, USA). Images were analyzed using Living Image 4.1 software (Caliper Life Sciences).

### Dual-luciferase reporter assay

Dual-luciferase reporter gene plasmids were purchased from GeneChem. Details are provided in the Supplementary Materials. HEK-293 T cells were seeded in 24-well plates and grown to 30% confluence 24 h before transfection. Cells were co-transfected with plasmid mixtures containing the 3′-untranslated region (3′-UTR) of genes (500 ng), and miRNA mimics or negative control (NC) (10 nM final concentration). After 48 h, activities of both firefly luciferase (LUC) and Renilla luciferase (RLUC) were measured with a Dual Luciferase Reporter Gene Assay Kit (Beyotime, Shanghai, China). LUC activity was normalized to RLUC activity to determine the ratio, and the fold-change was calculated by comparing the ratio from the miRNA mimic group to that of the NC group.

### Viability assays and colony formation assays

At 48 h post-transfection, cells were digested and seeded in 96-well plates (for Cell Counting Kit [CCK]-8 assays) or 6-well plates (for colony formation assays) at a density of 3 × 10^3^ or 1 × 10^3^ cells per well, respectively. For CCK-8 assays, at 0, 24, 48, and 72 h after seeding, the medium was replaced with 100 μL fresh DMEM containing 10% FBS and 10 μL CCK8 (Sigma-Aldrich, St. Louis, MO, USA) followed by incubation at 37 °C with 5% CO_2_ saturation for 1 h. The absorbance of the solution was measured at 450 nm using a Versamax microplate reader (Molecular Devices, Sunnyvale, CA, USA). For colony formation assays, 10 days after seeding, colonies were fixed with 4% paraformaldehyde for 30 min and stained with 1% crystal violet solution for another 10 min at 25 °C followed by image capture. Soft agar formation assays were conducted as previously reported [45].

### TCF/LEF activity assays

TOPFLASH plasmids (GeneChem) contain 6 TCF/LEF binding sites which are mutated in FOPFLASH plasmids. Stably transfected MG63 cells were seeded in 24-well plates at 40% confluence. After 24 h, MG63 cells were transiently transfected with 2 μg FOPFLASH or TOPFLASH plasmids, 0.2 μg Renilla luciferase plasmids. 48 h after transfection, activities of both firefly luciferase (LUC) and Renilla luciferase (RLUC) were measured with a Dual Luciferase Reporter Gene Assay Kit (Beyotime, Shanghai, China). The firely luciferase activity was normalized to the Renilla luciferase activity and expressed as the fold change compared with cells transfected with empty vector alone.

### Chromatin immunoprecipitation (ChIP) assay

The ChIP assay was performed according to the manufacturer’s recommendation (Cell Signaling Technology, US), with an average size of sheared fragments of about 200 to 1000 base pairs. In brief, 4 × 10^6^ to 6 × 10^6^ cells were treated with 1% formaldehyde to cross-link proteins to DNA. The chromatin was then prepared, sonicated and immunoprecipitated with indicated antibodies. The recovered DNA was detected by PCR using a primer flanking the LEF1-binding site at − 1804 in the human MYC promoter. The Sequence for primers is as follows: 5′-tgagagcaattaaagtagttagg-3′ (forward primer) and 5′-gcccagcatcttataattagtaa-3′ (reverse primer). The input sample contains 2% of the total input chromatin as PCR template in the detection of DNA pulled down with RUVBL1 and 0.5% of the total input in the detection of DNA pull downed with H4K16 (Acetylated). The band intensity of immunoprecipitated DNA in DNA-blot was normalized to that of input.

### Co-immunoprecipitation

MG63 cells were co-transfected with indicated plasmids with lipofectamine 3000 (Invitrogen). In brief, cells were lysed by RIPA Lysis Buffer (Cwbio) and lysates were incubated with indicated antibodies to capture the target protein at 4 °C overnight with gentle mixing. Next, lysates were incubated with Protein A/G agarose (Yeason, Shanghai, China) at 4 °C for 2-4 h so as to collect Protein A/G-Antibody-protein complexes. Protein A/G agarose was recollected via centrifuge and was washed to remove non-specifically bounded protein. The bounded protein was eluted with elution buffer for western blot analysis.

### Wound-healing assay

Transfected cells (1.5 × 10^5^ cells/well) were seeded in 6-well plates and incubated at 37 °C with 5% CO_2_ saturation overnight. Pipette tips (200 μl) were used to scrape a straight scratch in the confluent cell layer, and marker lines were drawn under the plate to mark the scratch position. Cells were washed twice with PBS, and images from specific scratched positions were captured by microscopy as a baseline. Fresh DMEM was added and, 24 h later, images from the same position were captured. The ratio of the area of wound-healing was quantified by ImageJ software (NIH, Bethesda, MD, USA).

### Transwell migration and invasion assay

At 48 h post-transfection, cells were digested, washed twice with PBS, re-suspended, and seeded in 12-well chamber-containing plates. Chambers for the invasion assay were pre-coated with Matrigel according to the manufacturer’s instructions (BD Science, Bedford, MA, USA). Serum-free DMEM (200 μl) containing cells (5 × 10^4^ cells/well for migration assay, 1 × 10^5^ cells/well for invasion assay) was added to the upper chamber. DMEM (300 μl) containing 10% FBS was added to the lower chamber. After 24 h, cells in the upper chamber were removed and the lower side of the chamber was gently washed twice with PBS and fixed with 4% paraformaldehyde for 30 min. Cells were then stained with 1% crystal violet solution for 10 min and images were captured by microscopy.

### Northern blot

The junction probe for circMYO10 was synthesized and labeled with digoxigenin. Northern blotting was performed as previously described [46]. Blots were washed stringently, detected by an anti-DIG antibody, and recorded on X-ray films with the chemiluminescence substrate CSPD (Roche).

### Immunofluorescence

Transfected cells at 70% confluence were fixed in 4% paraformaldehyde for 30 min and then permeabilized for 30 min in 0.5% Triton X-100. Cells were then blocked in 5% bovine serum albumin (BSA) for 1 h. Primary antibodies were diluted 1:100 in 5% BSA and incubated with cells overnight at 4 °C. The following day, the cells were gently washed several times in PBS, followed by incubation with CL594- or CL488-conjugated secondary antibodies (Proteintech Group, Rosemount, IL, USA) and diluted 1:300 in PBS for 1 h at 25 °C. Finally, cells were washed several times in PBS and immunofluorescence images were obtained using a Colibri epifluorescence microscope (Carl Zeiss, Jena, Germany) and processed with Image J software. Detailed information about the antibodies is provided in the Supplementary Materials.

### RNA fish

Cy3-labeled circMYO10 probes and fluorescein amidite-labeled miR-370-3p probes were designed and synthesized by RiboBio. A FISH Kit (RiboBio) was used to detect probe signals according to the manufacturer’s instructions. Nuclei were stained with 4,6-diamidino-2-phenylindole. All images were acquired on a LSM880 NLO (2 + 1 with BIG) confocal microscope system (Carl Zeiss).

### RNA immunoprecipitation

RNA immunoprecipitation (RIP) experiments were performed using the Magna RIP RNA-Binding Protein Immunoprecipitation Kit (Millipore, Billerica, MA, USA). MG63 cells stably expressing vector or shcircMYO10 were constructed. Approximately 1 × 10^7^ cells from each group were subjected to an equal pellet volume of RIP lysis Buffer (100 ml) plus protease inhibitors cocktail and RNase inhibitors. The cell lysates were incubated with IgG or anti-Ago2 antibody-coated beads (Millipore) and rotated at 4 °C overnight. The immunoprecipitated RNAs were extracted by a RNeasy MinElute Cleanup Kit (Qiagen, Valencia, CA, USA) after treatment with proteinase K buffer, and subjected to reverse transcription (CWBio). The circMYO10 levels were measured by quantitative reverse transcription PCR (qRT-PCR).

### Pull-down assay with biotinylated circMYO10 probe

OS cells (1 × 10^7^) were harvested, lysed, and sonicated. C-1 magnetic beads were incubated with circMYO10 probe (Life Technologies) at 25 °C for 2 h to generate probe-coated beads. The cell lysates were incubated with circMYO10 or oligo probe-coated C-1 magnetic beads to pull-down circMYO10. The RNA complexes bound to the beads were eluted and extracted with a RNeasy Mini Kit (Qiagen) for RT-PCR or real-time PCR. The biotinylated-circFAT1 probe was designed and synthesized by RiboBio.

### Microarray data

The full set of data of differentially expressed circular RNA, miRNAs, and genes in OS cells was downloaded from the NCBI Gene Expression Omnibus (GEO) as reported [44] and is accessible through the GEO Series accession numbers GSE96964, GSE28423, and GSE28424.

### Statistical analysis

Statistical analysis was carried out using GraphPad Prism 5.0 (GraphPad Software, La Jolla, CA, USA). Most graphs contain plots with each data point represented and the mean ± SD is shown. To test significance, t tests were performed and results are presented with asterisks indicating *P*-values.

## Results

### CircMYO10 is upregulated in human OS samples and cell lines

CircMYO10 was first reported in A549 and other cell lines [18] and was later found to be significantly upregulated in OS cell lines [44]. To the best of our knowledge, no further studies have been conducted on this circRNA to date.

**Fig. 1:**
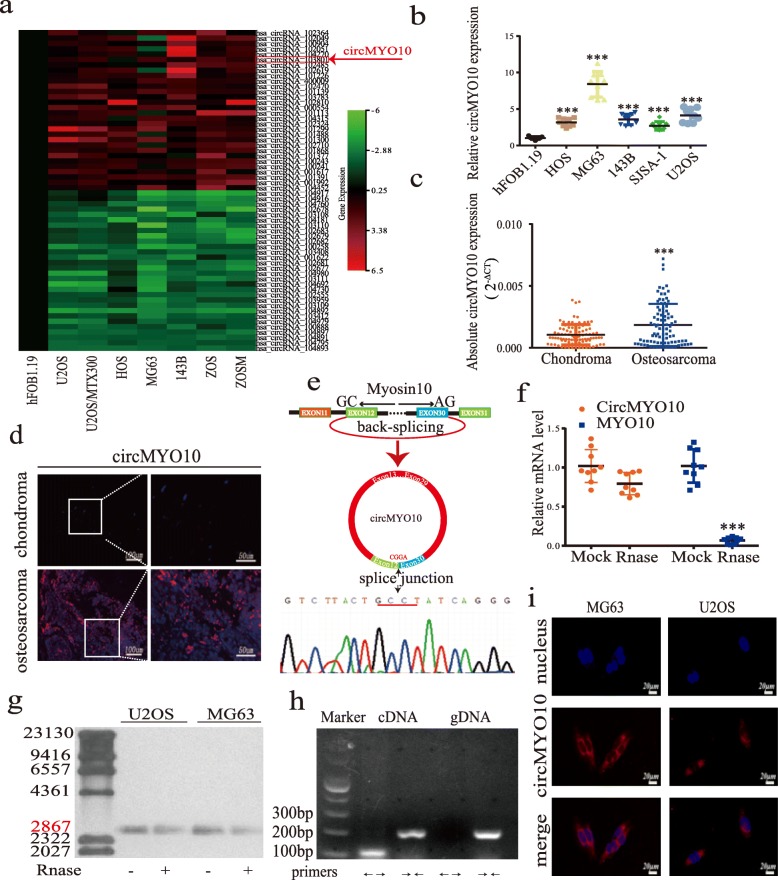
CircMYO10 validation and expression in osteosarcoma tissue and cells. **a** The heatmap for top 30 differentially up-and down-regulated circRNAs. **b** CircMYO10 expression in hFOB1.19 and osteosarcoma (OS) cell lines (143B, HOS, MG63, U2OS, SJSA-1) was evaluated by qRT-PCR. Data represents the mean ± standard deviation (SD) (*n* = 9). **c** CircMYO10 expression in ten paired human osteosarcoma tissues (*n* = 10) and chondroma tissues (*n* = 10) was measured by qRT-PCR. Data was normalized to the levels of GAPDH and presented as 2^-△CT^. Data represents the mean ± SD (*n* = 90). **d** RNA fluorescence in situ hybridization (FISH) showed that circMYO10 expression is higher in human OS tissues than in chondroma tissues. Representative images are shown. Scale bars = 100 μm or 50 μm. **e** Schematic illustration demonstrated the circularization of exons 12–30 of MYO10 forms circMYO10 by “head-to-tail” junction and the upper black arrow represents the splicing sites. The presence of circMYO10 was validated by RT-PCR followed by Sanger sequencing. **f** The expression of circMYO10 and MYO10 in MG63 cells treated with or without Rnase R was detected by qRT-PCR. The levels of circMYO10 and MYO10 mRNA in cells treated with Rnase R were normalized to the value measured in the mock treatment. Data represents the mean ± SD (*n* = 9). **g** Northern blots for detecting circMYO10 in U2OS and MG63 cells treated with or without RNase R digestion. The upper panels show the probed blots of circMYO10 with a red line indicating the band size of circMYO10 (2867 nt). The lower panels show the gel electrophoretic results of RNA with or without RNase R digestion. **h** Agarose gel electrophoresis found that divergent primers (←→) amplify circMYO10 in complementary DNA (cDNA) but not genomic DNA (gDNA). **i** FISH showed that circMYO10 localizes mainly in the cytoplasm. CirMYO10 probes were labeled with cy3 with nuclei stained with DAPI. Scale bars, 50 μm. Three independent assays were performed in the above assays. **b**, **c**, **f** * *P* < 0.05. ** *P* < 0.01. *** *P* < 0.001 (Student’s t-test)

We first analyzed the microarray data downloaded from GEO and drew a heatmap of the top 30 most differentially upregulated and downregulated circRNAs. This revealed the significant upregulation of hsa_cirRNA_103801 (circMYO10) in OS cell lines (Fig. 1a). To verify the RNA sequencing results, we detected circMYO10 expression in 10 paired chondroma and OS tissues as well as OS cell lines using qRT-PCR. CircMYO10 expression was upregulated in the OS cell lines, most significantly in U2OS and MG63 cells and in OS tissues (Fig. 1b-c). The observations were confirmed by RNA FISH (Fig. 1d). Divergent primers were designed and used to amplify the RT-PCR product of circMYO10, and Sanger sequencing confirmed the predicted back-splicing junction (Fig. 1e). CircMYO10 was very tolerant to the action of RNase R, while MYO10 mRNA levels were greatly decreased by exposure to RNase R (Fig. 1f). Next, we performed northern blot analysis for circMYO10 using total RNA extracted from MG63 and U2OS cells, and Rnase R was used to minimize the interruption of MYO10 mRNA (Fig. 1g). As shown in Fig. 1g, both cell lines (with or without RNase R digestion) showed a band of the expected size (2867 base pairs (bp)) using a digoxigenin-labeled circMYO10-specific probe targeting the junction region. We attempted to amplify circMYO10 from both genomic DNA and cDNA of MG63 cells using convergent and divergent primers. CircMYO10 was detected only from the cDNA while MYO10 was detected from both genomic DNA and cDNA (Fig. 1h). Moreover, FISH assays demonstrated that circMYO10 was mainly located in the cytoplasm (Fig. 1i).

### CircMYO10 knockdown inhibits OS progression in vitro

**Fig. 2:**
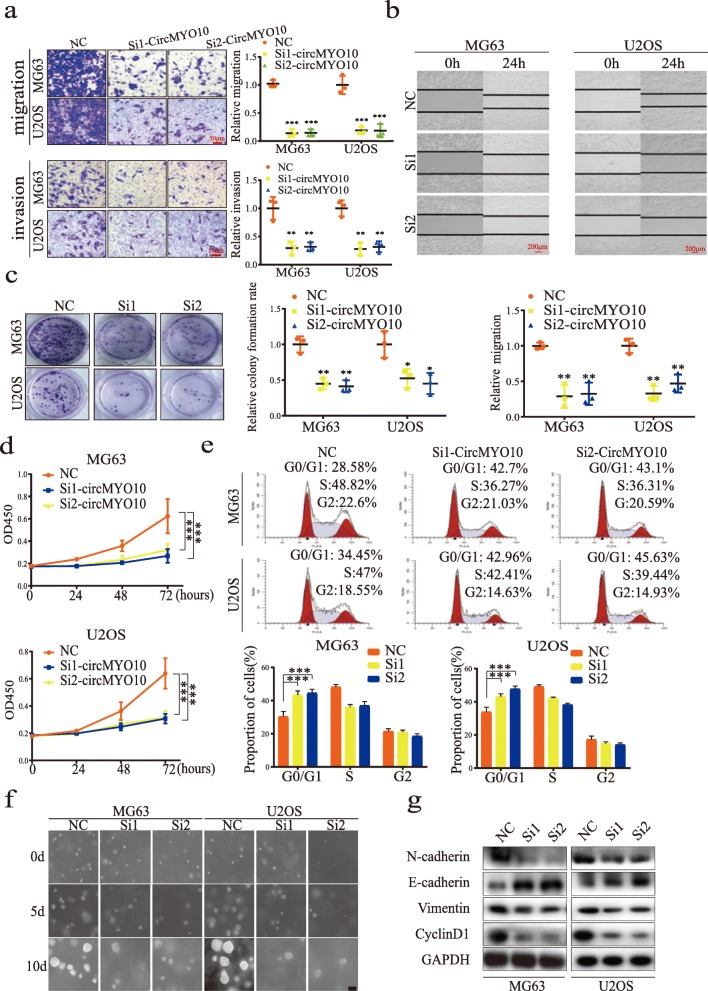
Knockdown of circMYO10 inhibits osteosarcoma cell proliferation and migration, and induces cell cycle arrest. **a** Transwell migration and invasion assays demonstrated that downregulation of circMYO10 compromises the cell migration and invasion abilities in MG63 and U2OS cells. Data represents the mean ± SD (*n* = 3). Scale bars = 50 μm. **b** CircMYO10 knockdown suppresses cell migration in MG63 and U2OS cells as evaluated by a wound healing assay. Data represents the mean ± SD (*n* = 3). Scale bars = 200 μm. **c** CircMYO10 knockdown inhibits colony formation in both MG63 and U2OS cells. Data represents the mean ± SD (*n* = 3). **d** Knockdown of circMYO10 inhibited cell proliferation as indicated by CCK-8 assays in MG63 and U2OS cells. Data represents the mean ± SD (*n* = 18). **e** Downregulation of circMYO10 arrested MG63 and U2OS cells at the G0/G1 phase. Data represents the mean ± SD (*n* = 3). **f** CircMYO10 knockdown inhibited anchorage-independent colony formation of both MG63 and U2OS cells. Scale bars = 50 μm. **g** The protein expression of N-cadherin, E-cadherin, Vimentin, and cyclinD1 was detected by western blot analysis in both MG63 and U2OS cells transfected with SicircMYO10. Three independent assays were performed in the above assays. **a**-**e** * *P* < 0.05, ** *P* < 0.01, *** *P* < 0.001 (Student’s t-test)

The observations of circMYO10 upregulation in OS tissues and cell lines prompted further investigation into its potential role. CircMYO10 expression was knocked down in MG63 and U2OS cells using siRNA transfection. Both siRNAs (si1- and si2-circMYO10), which target the junction site of circMYO10, significantly decreased circMYO10 expression as detected by qRT-PCR (Additional file 2: Figure S1a). The Transwell migration and invasion assay revealed that the migration and invasion capacities of MG63 and U2OS cells were impeded by circMYO10 inhibition (Fig. 2a). Silencing circMYO10 expression slowed the healing of scratches in the wound-healing assay, indicating that OS cell migration was suppressed (Fig. 2b). The colony formation assay showed that circMYO10 knockdown compromised cell proliferation which was consistent with the result of CCK-8 assays (Fig. 2c-d). Flow cytometry was conducted to determine whether the effect of circMYO10 knockdown on proliferation was due to alterations in the cell cycle. As shown in Fig. 2e, transfection with si-circMYO10 induced cell accumulation in the G1 phase, suggesting that circMYO10 led to G0/G1 cell cycle arrest. Moreover, anchorage-independent proliferation of MG63 and U2OS cells were inhibited upon circMYO10 knock down (Fig. 2f). EMT markers, Vimentin and N-cadherin, were significantly downregulated upon circMYO10 knockdown, while E-cadherin was markedly upregulated (Fig. 2g). Taken together, these results showed that silencing circMYO10 inhibits the proliferation and EMT of OS cells in vitro.

### In OS cells, CircMYO10 acts as a sponge for miR-370-3p

**Fig. 3:**
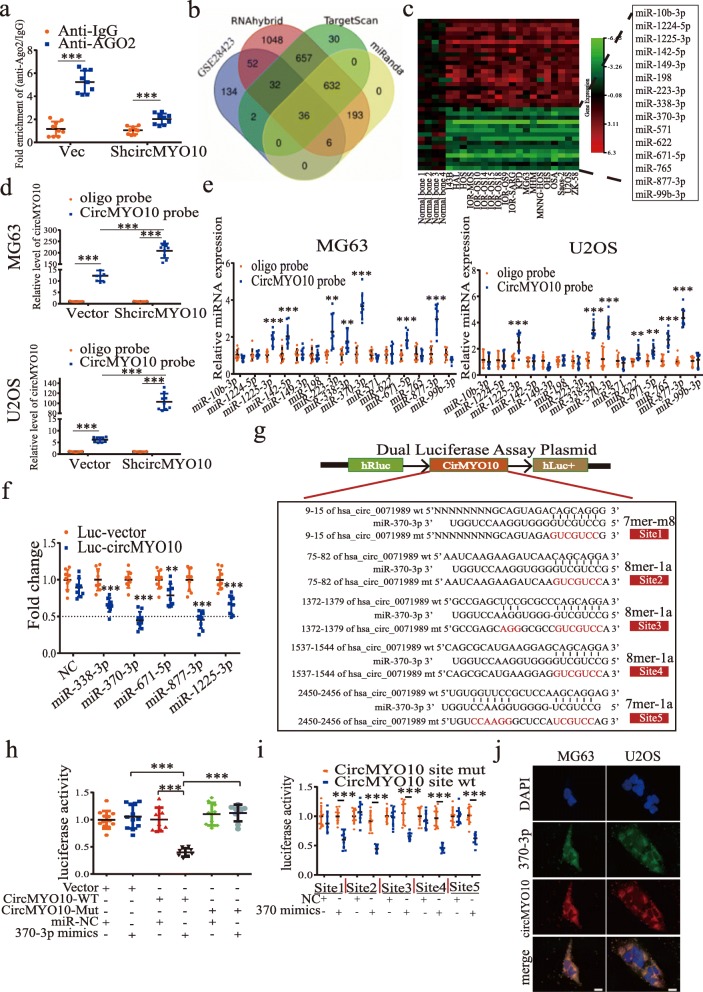
CircMYO10 acts as a sponge of miR-370-3p in osteosarcoma cells. **a** Ago2 RNA immunoprecipitation (RIP) assay for circMYO10 levels in MG63 cells stably expressing shcircMYO10. Data represents the mean ± SD (*n* = 9). **b** Using data from the GEO dataset GSE28423, differentially expressed miRNAs with |fold change| > 1 and *p* value < 0.05 were compared to the miRNAs common to the prediction of RNAhybrid, miRanda, and TargetScan that may bind to circMYO10. The Venn diagram shows the number of overlapping miRNAs. **c** The heat map for 36 differentially expressed miRNAs that may bind to circMYO10. **d** Lysates prepared from MG63 and U2OS cells stably transfected with circMYO10 or vector were subjected to RNA pull-down assays and were tested by qRT-PCR. Relative levels of circMYO10 pulled down by the circMYO10 probe were normalized to the level of circMYO10 pulled down by an oligo probe. Data represents the mean ± SD (*n* = 9). **e** The relative level of 15 miRNA candidates in the MG63 and U2OS lysates were detected by qRT-PCR. Data represents the mean ± SD (*n* = 9). **f**, **h**, **i** HEK-293 T cells were transfected with the indicated miRNA mimics and luciferase reporter plasmids. Forty-eight hours later, 293 T cells were lysed and the extracts were subjected to a dual-luciferase reporter assay. Data represent the mean ± SD (*n* = 9) for three independent experiments. **f** Luciferase activity of the Luc-vector or Luc-circMYO10 in 293 T cells co-transfected with indicated 5 miRNA mimics. **g** CircMYO10 was predicted to contain 5 putative sites for miR-370-3p. **h** 293 T cells were transfected with miR-370-3p mimics and Luciferase reporter plasmids containing either the wild type circMYO10 3′ UTR or a mutant circMYO10 3′ UTR where 5 binding sites for miR-370-3p were all mutated. **i** Each binding site, which was wild type or mutated, was cloned into luciferase reporter plasmids one by one. MiR-370-3p mimics were transfected into 293 T cells together with one of ten luciferase reporter plasmids separately. **j** FISH showed high co-localization of Cy3 labeled-circMYO10 and miR-370-3p labeled with Alexa Fluor 488 in MG63 and U2OS cells. Scale bars = 20 μm. Three independent assays were performed in the above assays. **a**, **d**-**f**, **h**-**i** * *P* < 0.05, ** *P* < 0.01, *** *P* < 0.001 (Student’s t-test)

CircRNAs act as miRNA sponges to regulate gene expression, which subsequently affects tumor progression [24]. Given the cytoplasmic location of circMYO10, we hypothesized that circMYO10 may act as a miRNA sponge to regulate OS progression. To evidence our hypothesis, we applied RNA immunoprecipitation in MG63 cells and found that circMYO10 was pulled down with Ago2 and was less enriched in the group where MG63 cells were stably transfected with shcircMYO10 compared with cells transfected with empty vector (Fig. 3a). To confirm that circMYO10 acts as a sponge in MG63 and U2OS cells, we used RNAhybrid, miRanda and TargetScan algorithms to predict potential miRNAs that bound to circMYO10. The result revealed 668 miRNAs that were common to all three outputs. To narrow the range of targets, we downloaded dataset GSE28423 in the GEO database and compared differentially expressed miRNAs, with |fold change| > 1 and *p* value < 0.05, to the 668 miRNAs predicted by three algorithms and found that 36 miRNAs matched the condition (Fig. 3b). As shown in Fig. 3c, 21 miRNAs were upregulated in OS and 15 downregulated. Since inhibiting circMYO10 suppressed OS cells progression, we designed a biotin-labeled circMYO10 probe and 15 downregulated miRNAs were subjected to a pull-down assay to investigate whether those miRNAs bound to circMYO10 directly. As shown in Fig. 3d, circMYO10 overexpression significantly increased the amount of circMYO10 pulled down by the probe. The expression of RNA eluted after the pull-down assay was detected by qRT-PCR. As illustrated in Fig. 3e, miR-338-3p, miR-370-3p, miR-671-5p, miR-877-3p, and miR-1225-3p were more enriched in RNAs pulled down by the circMYO10 probe compared to RNAs pulled down by an oligo probe in both cell lines. Next, A luciferase reporter gene containing the full-length circMYO10 sequences was constructed. MiR-370-3p and miR-877-3p strongly reduced luciferase activity more than 50% compared with control (Fig. 3f). Moreover, we predicted the seed regions between circMYO10 and either miR-370-3p or miR-877-3p. CircMYO10 contains three 8mer-1a, one 7mer-m8, and one 7mer-1a potential targets of miR-370-3p and three 8 mer-1a targets of miR-877-3p (Fig. 3g and Additional file 3: Figure S2a). Next, we compared the effect of miR-370-3p and miR-877-3p on the migration, invasion and EMT ability of MG63 and U2OS cells. Interestingly, miR-370-3p showed a stronger effect than miR-877-3p in EMT and we focused much more on the research into the role of miR-370-3p (Additional file 4: Figure S3a-b). To further verify the interaction between circMYO10 and miR-370-3p, we constructed a luciferase reporter gene where all 5 sites were mutated. When transfected with miR-370-3p mimics, reporter plasmids containing mutant circMYO10 3′ UTR showed no significant effect on luciferase activity compared to those transfected with wild reporter genes containing wild type circMYO10 3′ UTR (Fig. 3h). Surprisingly, when cloned into luciferase reporter genes one by one, the 5 binding sites were all verified to be functional with sites 2 and 4 reducing the luciferase activity to the greatest extent (Fig. 3i). The RNA FISH assay revealed a high degree of co-localization between circMYO10 and miR-370-3p in MG63 and U2OS cells (Fig. 3j). These results suggested that circMYO10 acts as a sponge for miR-370-3p.

### MiR-370-3p suppresses OS cells proliferation and EMT

**Fig. 4:**
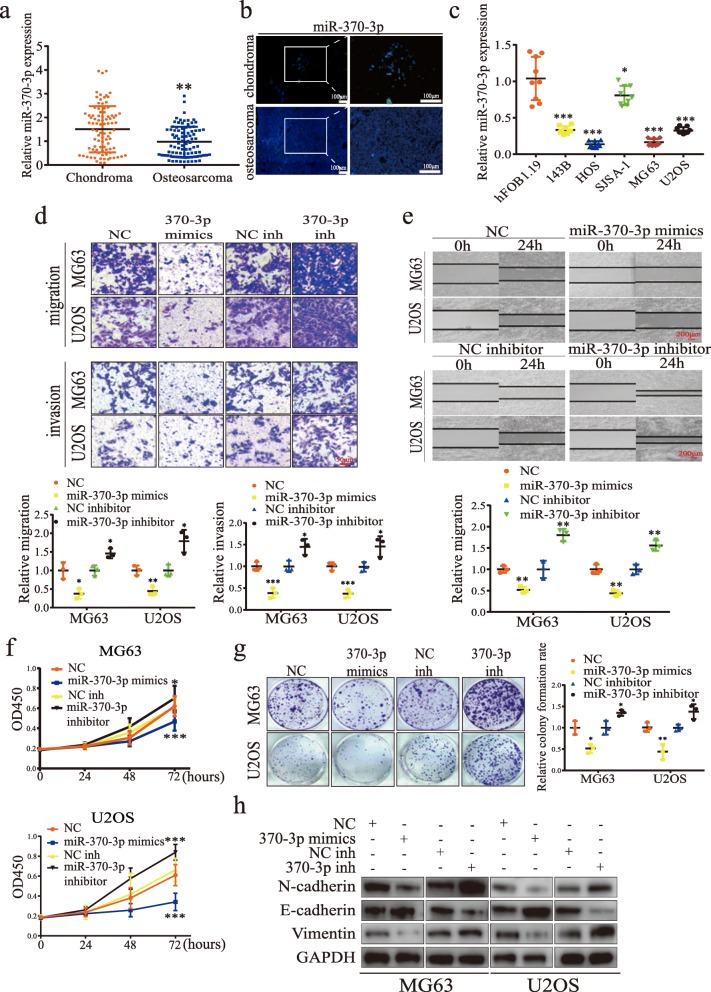
MiR-370-3p inhibits osteosarcomas cells proliferation and EMT. **a** The expression of miR-370-3p in ten paired chondroma (*n* = 10) and osteosarcoma tissues (*n* = 10) was measured by qRT-PCR and FISH assays. **a** Data represents the mean ± SD (*n* = 90 per group). **b** FISH assays showed miR-370-3p expression is lower in human OS tissue than in chondroma tissue. Representative images are shown. Scale bars = 100 μm. **c** The expression of miR-370-3p in hFOB1.19, 143B, U2OS, HOS, MG63, and SJSA-1 was measured by qRT-PCR. Data represents the mean ± SD (*n* = 9). **d** The effect of miR-370-3p on migration and invasion was measured in Transwell migration and invasion assays. Data represents the mean ± SD (*n* = 3). Scale bars =50 μm. **e** The effect of miR-370-3p overexpression and inhibition on migration was measured by wound healing assays. Data represents the mean ± SD (*n* = 3). Scale bars = 200 μm. **f** Proliferation of cells transfected with miR-370-3p mimics or miR-370-3p inhibitor was measured by CCK-8 assay in MG63 and U2OS cells. Data represents the mean ± SD (*n* = 18). **g** Downregulation of miR-370-3p stimulates colony formation and overexpression of miR-370-3p suppresses colony formation in MG63 and U2OS cells. Data represents the mean ± SD (*n* = 3). **f** The effect of miR-370-3p on the expression of CyclinD1, E-cadherin, N-cadherin, and vimentin was detected by western blot analysis. Three independent assays were performed in the above assays. **a**, **c**, **d**-**f** * *P* < 0.05, ** *P* < 0.01, *** *P* < 0.001 (Student’s t-test)

The role of miR-370-3p in suppressing tumor-associated genes has been described in multiple cancer types, but its role in OS has rarely been described [47–50]. To investigate the role of miR-370-3p in OS, we first detected the expression of miR-370-3p in human OS tissues and OS cell lines. As revealed in Fig. 4a-c, miR-370-3p was downregulated in OS tissues when compared to chondroma tissues, and was downregulated in 143B, HOS, SJSA-1, MG63 and U2OS cells when compared to hFOB1.19 cells. To study the function of miR-370-3p, miR-370-3p mimics and inhibitors were separately transfected into MG63 and U2OS cells and their effect on the expression of miR-370-3p was detected (Additional file 2: Figure S1b). As has been reported in thyroid cancer [50], miR-370-3p overexpression inhibited the ability of cells to migrate and invade, whereas its downregulation had the opposite effect (Fig. 4d). Similar results were obtained for the effect of miR-370-3p on migration as manifested in our wound-healing assays (Fig. 4e). The CCK-8 assay revealed that miR-370-3p mimics compromised cell viability, while their inhibitors led to an increase in cell proliferation (Fig. 4f). In addition, plate colony formation assays demonstrated that the colony-forming potential of cells was compromised or promoted upon treatment with miR-370-3p mimics or miR-370-3p inhibitors, respectively, as compared with that in the NC group (Fig. 4g). Western blot analysis showed that overexpressing miR-370-3p downregulated the expression of N-cadherin and Vimentin, and upregulated E-cadherin; miR-370-3p inhibition produced the reverse result, indicating the inhibitory effect of miR-370-3p on EMT (Fig. 4h). Collectively, the results suggested that miR-370-3p inhibits the proliferation and EMT of OS cells in vitro.

### RUVBL1 is a direct target of miR-370-3p and is an oncogene involved in Wnt/β-catenin signaling in osteosarcoma

**Fig. 5:**
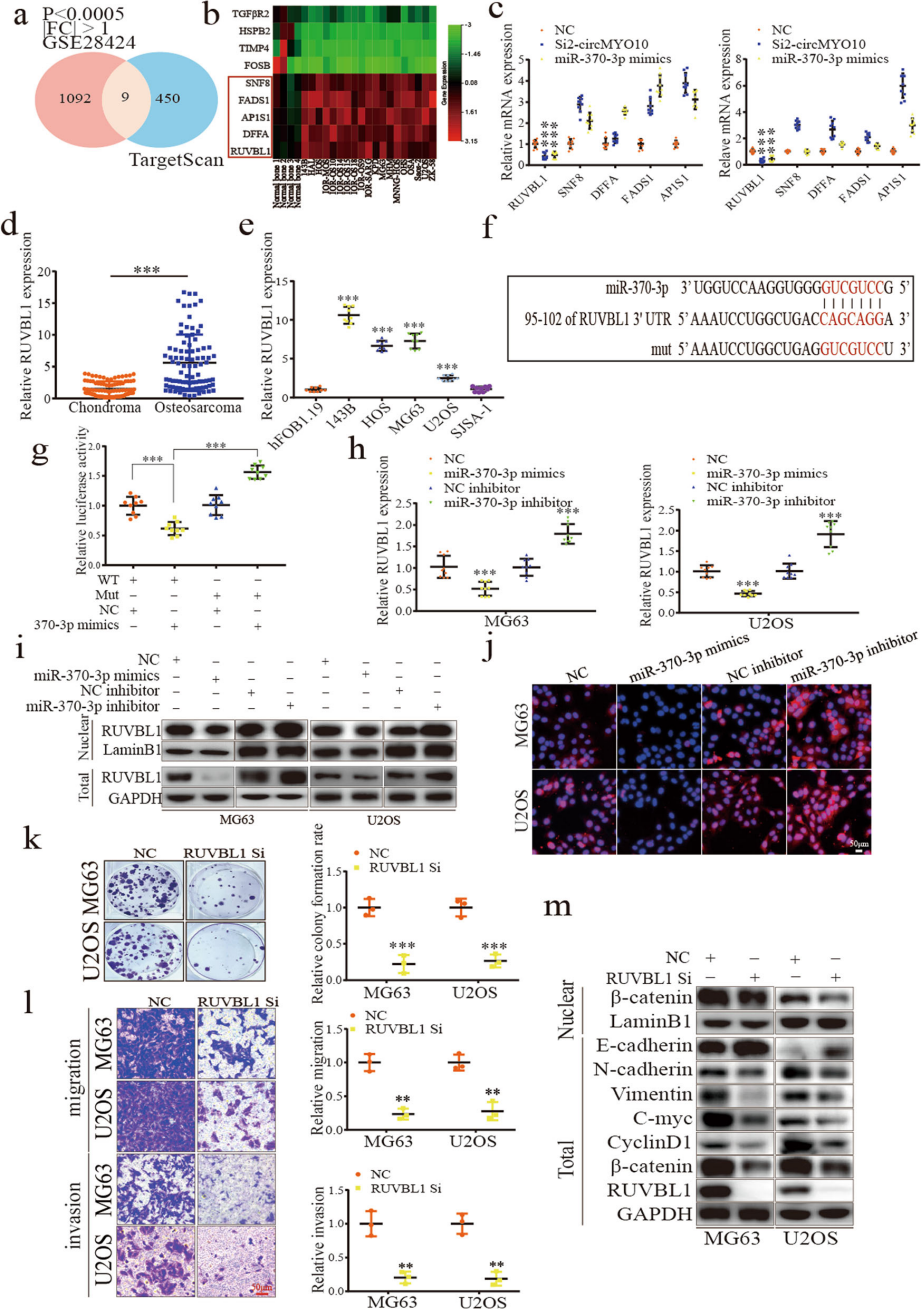
RUVBL1 is a direct target of miR-370-3p and an oncogene in OS involved in Wnt/β-catenin signaling. **a** Target genes of miR-370-3p were predicted by TargetScan and compared with differentially expressed genes in the GEO dataset GSE28424. Overlapped genes matching the condition where |fold change| > 1 and *p* value < 0.0005 were chosen. **b** The heat map showed the 4 differentially downregulated and 5 upregulated target genes of miR-370-3p. **c** The upregulated 5 genes were subjected to qRT-PCR in MG63 cells and U2OS cells transfected with either Si2-circMYO10 and miR-370-3p mimics. Data represents the mean ± SD (*n* = 9). **d**-**e** The abundance of RUVBL1 in 10 paired chondroma (*n* = 10), osteosarcoma tissues (*n* = 10), hFOB1.19 and osteosarcoma cell lines was detected by qRT-PCR. **d** Data represents the mean ± SD (*n* = 90). **e** Data represents the mean ± SD (*n* = 9). **f** The putative binding site of the RUVBL1 3′ UTR for miR-370-3p. **g** The luciferase reporter plasmids containing wild type or mutated RUVBL1 3′ UTRs were con-transfected with miR-370-3p mimics into 293 T cells. Data represents the mean ± SD (*n* = 9). **i**-**j** The expression of RUVBL1 in MG63 cells transfected with control or miR-370-3p mimics or miR-370-3p inhibitors was detected by **h** qRT-PCR, **i** western blot and **j** immunofluorescence analysis. **h** Data represents the mean ± SD (*n* = 9). **k** RUVBL1 knockdown inhibited the colony formation ability of both MG63 and U2OS cells. Data represents the mean ± SD (*n* = 3). **l** Compromised migration and invasion ability were detected in MG63 and U2OS cells with RUVBL1 inhibited. Scale bars = 200 μm. Data represents the mean ± SD (*n* = 3). **m** Western blot analysis indicating the inhibited Wnt/β-catenin signaling as evidenced by the downregulation of C-myc, CyclinD1, β-catenin, nuclear β-catenin, N-cadherin and Vimentin with E-cadherin upregulated. Three independent assays were performed in the above assays. **c**-**e**, **g**-**h** * *P* < 0.05, ** *P* < 0.01, *** *P* < 0.001 (Student’s t-test)

To further study the function of miR-370-3p in OS, we first predicted the potential targets of miR-370-3p using the TargetScan algorithm. To find potential oncogenic genes, we compared potential target genes with the datasets from GSE28424 and selected genes which are differentially expressed in OS with |fold change| > 1 and *p* value < 0.0005 (Fig. 5a). As shown in Fig. 5b five genes were shown to be the target of miR-370-3p and were significantly upregulated in OS (Fig. 5b). Next, Si2-circMYO10 and miR-370-3p mimics were transfected into either MG63 or U2OS cells separately and qRT-PCR was applied. Upon either circMYO10 inhibition or miR-370-3p overexpression, the mRNA level of RUVBL1 was the only one downregulated which prompted the further investigation of RUVBL1 (Fig. 5c). Next, it was shown that RUVBL1 was significantly upregulated in human OS tissues than in chondroma tissues (Fig. 5d). Consistent with the result of RNA sequences, RUVBL1 was highly expressed in OS cell lines including 143B, HOS, MG63 and U2OS (Fig. 5e). As illustrated in Fig. 5f, the RUVBL1 3′ UTR contains an 8mer-1a site for miR-370-3p (Fig. 5f). To investigate whether miR-370-3p binds to the RUVBL1 3′ UTR, we applied a dual luciferase reporter assays and found that miR-370-3p mimics significantly reduced the luciferase activity of reporter genes containing RUVBL1 3′ UTR when compared with NC, and the reduction was abrogated when the binding site in RUVBL1 3′ UTR for miR-370-3p was mutated (Fig. 5g). Moreover, protein and mRNA levels of RUVBL1 were both significantly downregulated by miR-370-3p mimics and was upregulated by miR-370-3p inhibitors as evidenced by qRT-PCR, western blot and immunofluorescence analysis (Fig. 5h-j). These results indicated that RUVBL1 is a direct target of miR-370-3p.

RUVBL1 has been reported as an oncogene in various cancers, but its role in OS remains unknown [51, 52]. As RUVBL1 was previously reported to positively regulate Wnt/β-catenin signaling [39, 40], we wondered whether RUVBL1 regulates the Wnt/β-catenin signaling pathway to promote OS progression. MG63 and U2OS cells were transfected with RUVBL1 siRNA to study the function of RUVBL1 in OS. Interestingly, RUVBL1 suppression resulted in extremely strong inhibition of cell proliferation (Additional file 5: Figure S4a). Consistent with the result of CCK-8 assays, RUVBL1 inhibition compromised both the anchorage-dependent and -independent colony formation ability of transfected cells (Fig. 5k and Additional file 5: Figure S4b). Furthermore, wound-healing assays also showed slower scratch healing speeds for cells transfected with RUVBL1 siRNA (Additional file 5: Figure S4c). With RUVBL1 inhibited, significantly fewer cells were shown to migrate and invade through transwells (Fig. 5l). Moreover, the expression of C-myc, CyclinD1 and β-catenin was significantly downregulated in cells with RUVBL1 inhibited (Fig. 5m and Additional file 6: Figure S5a-b). Importantly, nuclear β-catenin, which is required for the activation of canonical Wnt/β-catenin signaling, was downregulated upon RUVBL1 inhibition (Fig. 5m). In addition, depression of RUVBL1 upregulated the expression of E-cadherin, contrary to the result of N-cadherin and Vimentin (Fig. 5m). While the cytoplasmic location of RUVBL1 may be involved in EMT and cancer metastasis [53, 54], we found that RUVBL1 is located both in the cytoplasm and nucleus in MG63 and U2OS cells (Additional file 6: Figure S5b). These results showed that RUVBL1 is an oncogene involved in Wnt/β-catenin signaling and EMT in OS and is also a direct target of miR-370-3p.

Via the inhibition of miR-370-3p, circMYO10 upregulates RUVBL1 expression to promote the interaction between RUVBL1 and β-catenin/LEF1 complex and thus promotes Wnt/β-catenin signaling.

It was reported that RUVBL1 promotes Wnt/β-catenin signaling via formation of RUVBL1/β-catenin/TCF4 complex [39, 40, 42, 43, 55]. Given that RUVBL1 is a direct target of miR-370-3p, next, we sought to investigate the role of miR-370-3p in Wnt/β-catenin signaling and whether miR-370-3p inhibits the interaction between RUVBL1 and β-catenin/LEF1 complex.

**Fig. 6:**
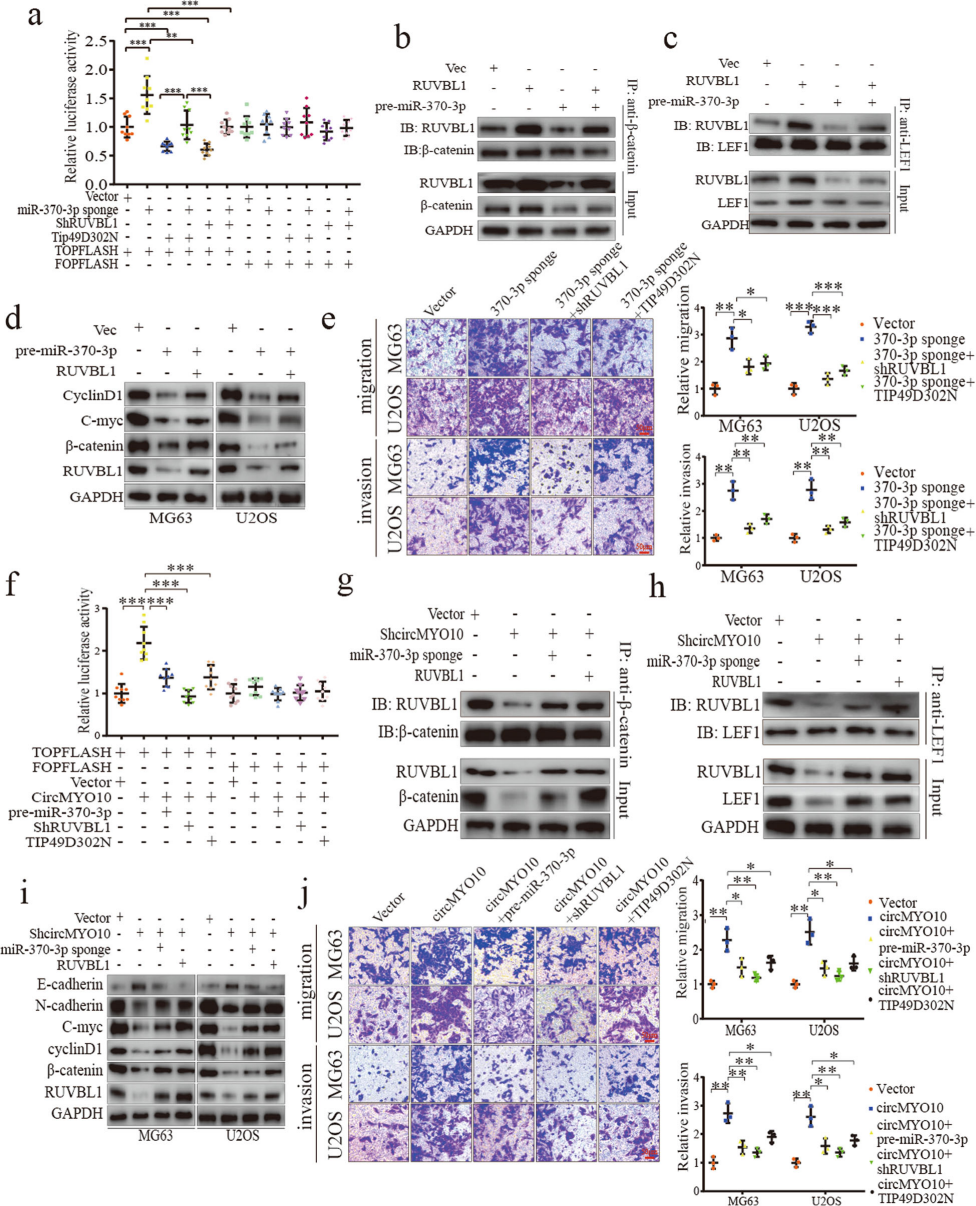
CircMYO10 inhibits miR-370-3p to upregulate RUVBL1 expression and promotes the interaction between RUVBL1 and β-catenin/LEF1 complex and thus activates Wnt/β-catenin signaling. **a** MG63 cells were stably transfected with plasmids indicated while TOP/FOPFLASH plasmids, together with Renilla luciferase plasmids, were transiently transfected to measure the luciferase activity. Renilla luciferase activity was used as an internal control. Data represents the mean ± SD (*n* = 9). **b**-**c** MG63 cells were stably transfected with expression vectors as indicated. Pre-miR-370-3p reduced the expression of RUVBL1 and β-catenin in MG63 cells. Lysates were incubated with either anti-β-catenin or anti-LEF1. Immunoprecipitated complexes were subjected to western blot analysis. Anti-RUVBL1 antibodies derived from different species were used to avoid the detection of heavy chains. **d** Western blot analysis of RUVBL1, β-catenin, C-myc and CyclinD1 in MG63 cells and U2OS cells stably transfected expression vectors as indicated. **e** Transfection with miR-370-3p sponge promoted the migration and invasion ability of both MG63 and U2OS cells which was partially abrogated by either ShRUVBL1 or TIP49D302N. Data represents the mean ± SD (*n* = 3). (f) TCF/LEF luciferase activity assays were conducted in MG63 cells transfected with expression vectors indicated. Data represents the mean ± SD (*n* = 9). **g**-**h** ShcircMYO10 downregulated the expression of **g** β-catenin, **g** RUVBL1 and **h** LEF1, which was restored by co-transfection with either RUVBL1 or miR-370-3p sponge in MG63 cells. Proteins immunoprecipitated with β-catenin either LEF1 were subjected to western blot analysis. **i** ShcircMYO10 decreases the expression of RUVBL1, β-catenin, cyclinD1, C-myc, N-cadherin and vimentin with E-cadherin upregulated, and the change was partially attenuated by co-transfection with either miR-370-3p sponge or RUVBL1. **j** The enhanced migration and invasion ability of MG63 and U2OS cells were partially abrogated by co-transfection with any of Pre-miR-370-3p or shRUVBL1 or TIP49D302N. Data represents the mean ± SD (*n* = 3). Three independent assays were performed in the above assays. **a**, **e**, **f**, **j** * *P* < 0.05, ** *P* < 0.01, *** *P* < 0.001 (Student’s t-test)

TIP49D302N (D302 → N in the walker B box), a negative form of RUVBL1 without ATPase activity but still competing with wild type RUVBL1 for β-catenin [42, 43], was used to investigate the biological relevance between miR-370-3p and RUVBL1. MiR-370-3p sponge, shRUVBL1 and TIP49D302N plasmids were transfected solely or co-transfected selectively into MG63 cells to construct stable cell lines. By applying TCF/LEF activity assay, we found that TCF/LEF activity was significantly increased in cells stably transfected with miR-370-3p sponge, indicating the inhibitory role of miR-370-3p in Wnt/β-catenin signaling which was also evidenced by the result of our qRT-PCR, western blot, immunofluorescence analysis of C-myc, cyclinD1 and β-catenin expression (Fig. 6a and Additional file 7: Figure S6a-c); TCF/LEF activity was decreased in cells transfected with either shRUVBL1 or TIP49D302N when compared with control group (Fig. 6a). Moreover, the enhancement induced by miR-370-3p sponge was partially attenuated in cells transfected with miR-370-3p sponge and shRUVBL1 or miR-370-3p sponge and TIP49D302N, which may indicate the involvement of RUVBL1 in the process where miR-370-3p inhibits Wnt/β-catenin signaling, and that the interaction between RUVBL1 and β-catenin, which was interrupted by TIP49D302N, was also involved (Fig. 6a). To investigate directly, we applied co-immunoprecipitation assays in stably transfected MG63 cells. Since the band size of RUVBL1 was pretty close to that of heavy chains, antibodies from different species were used for immunoprecipitation and immunoblot. As shown in Fig. 6b, we found that pre-miR-370-3p downregulated the expression of RUVBL1 and β-catenin. Interaction between β-catenin and RUVBL1 was inhibited in cells transfected with pre-miR-370-3p when the amount of protein was kept constant (Fig. 6b). Since the enhanced transcription of Wnt target genes requires the involvement of TCF/LEF, we wonder whether the presence of RUVBL1 in RUVBL1/β-catenin/LEF1 complex was also reduced. In cells transfected with pre-miR-370-3p, while the expression of LEF1 was relatively constant, we found that less RUVBL1 was immunoprecipitated with LEF1, which was consistent with the result in input indicating that the reduced interaction was largely due to the decreased expression induced by pre-miR-370-3p (Fig. 6c). Overexpression of RUVBL1 in cells transfected with pre-miR-370-3p restored the downregulated expression of RUVBL1 and the interaction between RUVBL1 and LEF1 (Fig. 6c). The upregulation of β-catenin induced by RUVBL1 in miR-370-3p-overexpressed cells may be due to the restored Wnt/β-catenin signaling since the inhibited expression of Wnt target genes C-myc and CyclinD1 caused by miR-370-3p was also attenuated (Fig. 6d). Next, we investigated whether RUVBL1 is able to reverse the phenotype that miR-370-3p induces. As revealed in Fig. 6e, we found that either shRUVBL1 or TIP49D302N inhibited the enhanced migration and invasion ability of both MG63 and U2OS cells caused by miR-370-3p sponge (Fig. 6e). Collectively, these results showed that miR-370-3p downregulates the expression of RUVBL1 and inhibits it from the formation of RUVBL1/β-catenin/LEF1 complex and thus inhibits Wnt/β-catenin signaling to suppress OS progression.

Since we had shown that circMYO10 acts as a sponge for miR-370-3p, we wondered whether circMYO10 is involved in the regulation of Wnt/β-catenin signaling via miR-370-3p/RUVBL1 axis. By applying TCF/LEF activity assats, we found that TCF/LEF activity was significantly increased in circMYO10 group when compared to Vector group (Fig. 6f). Moreover, transfection with any of miR-370-3p sponge, shRUVBL1 or TIP49D302N in circMYO10-overexpressed MG63 cells restored the augmented TCF/LEF activity, which revealed the possibility that miR-370-3p/RUVBL1 axis may mediate the process where circMYO10 promotes Wnt/β-catenin signaling (Fig. 6f). Next, we investigated whether circMYO10 promotes the interaction between RUVBL1 and β-catenin via miR-370-3p. Interestingly, we found that circMYO10 inhibition downregulated the expression of both β-catenin and RUVBL1; less RUVBL1 was immunoprecipitated with β-catenin when the amount of protein was kept relatively constant (Fig. 6g). Co-transfection with either miR-370-3p sponge or RUVBL1 attenuated the decreased expression of β-catenin and RUVBL1 in input and restored the interaction between β-catenin and RUVBL1 indicating that circMYO10 promotes the interaction between RUVBL1 and β-catenin partially through the inhibition of miR-370-3p (Fig. 6g). Next, we investigated whether circMYO10 upregulates the expression of RUVBL1 to promote the interaction between RUVBL1 and β-catenin/LEF1 complex via inhibition of miR-370-3p. Besides RUVBL1, the expression of LEF1 was also downregulated in circMYO10-inhibited MG63 cells (Fig. 6h). Either miR-370-3p sponge or RUVBL1 restored the downregulated expression of RUVBL1 and LEF1 as well as the amount of RUVBL1 immunoprecipitated with LEF1 (Fig. 6h). Next, we investigated whether the expression of Wnt target genes was consistent with the interaction between RUVBL1 and β-catenin/LEF1 complex. As a result, knockdown of circMYO10 downregulated the expression of RUVBL1, β-catenin, C-myc, cyclinD1, together with EMT markers N-cadherin and Vimentin while E-cadherin was upregulated (Fig. 6i). Moreover, the induced effect by shcircMYO10 was partially neutralized by co-transfection with miR-370-3p sponge or RUVBL1 (Fig. 6i). Consistent with the related Wnt/β-catenin signaling, shcircMYO10 inhibited MG63 and U2OS cells to migrate and invade, and the compromised ability was partially restored by co-transfection with either miR-370-3p sponge or RUVBL1 (Fig. 6j). Similar results, showing the involvement of RUVBL1 and miR-370-3p in circMYO10-induced phenotypes, were also obtained in our CCK-8 and colony formation, cell cycle analysis (Additional file 8: Figure S7a-c). Collectively, those results showed that circMYO10 upregulates the expression of RUVBL1 to promote the interaction between RUVBL1 and β-catenin/LEF1 complex and activates Wnt/β-catenin signaling via the inhibition of miR-370-3p, which further accelerates OS progression.

### CircMYO10 promotes histone H4K16 acetylation in the promoter region of C-myc partially through miR-370-3p/RUVBL1 axis

Since reduced interaction between RUVBL1 and β-catenin/LEF1 complex was not sufficient to explain the reduced Wnt/β-catenin signaling, we sought to find further mechanistic insight into circMYO10/miR-370-3p/RUVBL1 axis.

**Fig. 7:**
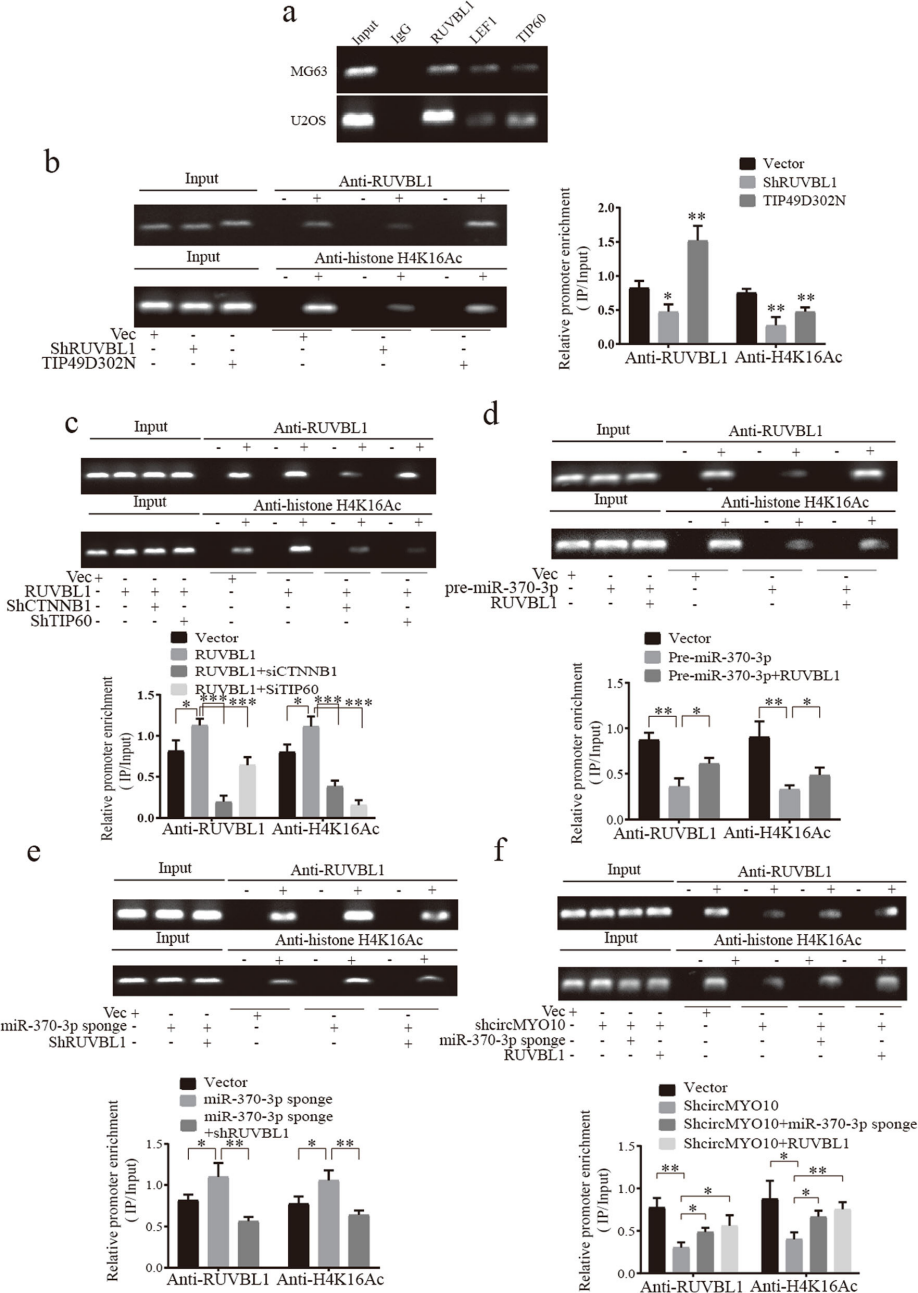
CircMYO10 promotes H4K16Ac at the promoter region of C-myc via miR-370-3p/RUVBL1 axis. **a** The occupancy of the MYC promoter by RUVBL1, LEF1, TIP60. **a**-**f** Chromatin was collected from MG63 and U2OS cells and was subjected to immunoprecipitation with IgG or anti-LEF1-antibodies or anti-TIP60 antibodies or anti-RUVBL1 antibodies or anti-histone H4K16Ac. Recovered DNA was used as a template in PCR amplification using the pair of primers flanking the LEF1-binding site at − 1804. 2% Input was used as a control in the detection of DNA immunoprecipitated with RUVBL1, LEF1 and TIP60 with 0.5% input for DNA immunoprecipitated with H4K16Ac. **b**-**c** The recruitment of RUVBL1 to the promoter region and measurement of H4K16Ac by CHIP assays in MG63 cells stably transfected with either **b** shRUVBL1 or **b** TIP49D302N or **c** RUVBL1 or **c** shCTNNB1 or **c** shTIP60. **d** MG63 cells stably transfected with pre-miR-370-3p inhibited the recruitment of RUVBL1 to the promoter region and H4K16Ac, both of which was restored by co-transfection with RUVBL1. **e** MiR-370-3p sponge promoted the recruitment of RUVBL1 and H4K16Ac which was abrogated by shRUVBL1. **f** Recruitment of RUVBL1 to the promoter region was suppressed upon transfection with shcircMYO10 but partially restored by co-transfection with either miR-370-3p sponge or RUVBL1 in MG63 cells. The acetylation of H4K16 was highly correlated with the recruitment of RUVBL1. **b**-**f** Data represents the mean ± SD (*n* = 3). Three independent assays were performed in the above assays. **b**-**f** * *P* < 0.05, ** *P* < 0.01, *** *P* < 0.001 (Student’s t-test)

Previous study showed that RUVBL1 is a coactivator of TIP60 and enhances the transcription activity of TCF4 via histone H4 acetylation and thus the expression of ITF-2, a Wnt target gene [42]. More specifically, recent study has shown that methylated RUVBL1 promotes H4K16 acetylation and thus the removal of 53BP1 to enhance the activity of TIP60 contributing to DNA repair [56]. Since H4K16 acetylation (H4K16Ac) is also considered important for active transcription and maintenance of euchromatin [57], we wondered whether RUVBL1 enhances the transcription activity of β-catenin/LEF1 via its effect on H4K16Ac, and the LEF1 binding site in the promoter region of C-myc was selected for CHIP assays for reasons that C-myc is not only an extremely important oncogene but also regulated by Wnt/β-catenin signaling. Interestingly, RUVBL1 was detected at the LEF1 binding site, located at − 1804 in the promoter region of C-myc, together with LEF1 and Tip60 (Fig. 7a), while TIP60 was previously reported as a co-activator of C-myc to promote its transcription activity via chromatin acetylation [58]. To investigate the role of RUVBL1 in H4K16Ac, MG63 cells were stably transfected with either vector or shRUVBL1 or TIP49D302N. As revealed in Fig. 7b, the amount of RUVBL1 recruited to the promoter region was reduced in cells transfected with shRUVBL1 and increased in cells transfected with TIP49D302N. Moreover, H4K16Ac in the indicated region of C-myc was inhibited in MG63 cells transfected with either shRUVBL1 or TIP49D302N (Fig. 7b), both of which indicated that RUVBL1 enhanced H4K16Ac. On the other side, we found that overexpression of RUVBL1 in MG63 cells also promoted H4K16Ac in the indicated region (Fig. 7c). However, RUVBL1 failed to be recruited at the promoter region of C-myc even in RUVBL1-overexpressed cells, accompanied by the inhibited H4K16Ac, when β-catenin was knocked down (Fig. 7c). Given that direct interaction between β-catenin and RUVBL1 was widely reported [39, 40], that interrupted interaction by TIP49D302N inhibited H4K16Ac in the promoter region and that knockdown of β-catenin inhibited the recruitment of RUVBL1 to the promoter region of C-myc (Fig. 7c), it was strongly suggested that β-catenin is required for the role of RUVBL1 in Wnt/β-catenin signaling via its effect on H4K16Ac. Consistent with the result as previously reported [56], the H4K16Ac-related process in the regulation of C-myc was at least mediated by TIP60 since we found that knockdown of TIP60 in RUVBL1-overexpressed cells almost totally abrogated H4K16Ac in the indicated region while there was only a moderate decrease in the enrichment of RUVBL1 at the promoter region when compared to RUVBL1-overexpressed group (Fig. 7c). Our results, along with the role of RUVBL1 as previously reported [39, 40, 42, 55–57], suggested that RUVBL1 is recruited by β-catenin, which made the interaction between β-catenin and RUVBL1 necessary for the role of RUVBL1 in Wnt/β-catenin signaling, and RUVBL1 enhances the transcription activity of LEF1 to induce C-myc expression partially by promoting H4K16Ac which was at least mediated by TIP60.

Next, we sought to investigate whether circMYO10 and miR-370-3p had a role in H4K16Ac via RUVBL1. First, we transfected MG63 cells with vector, pre-miR-370-3p or pre-miR-370-3p and RUVBL1. Interestingly, as illustrated in Fig. 7d, pre-miR-370-3p decreased the amount of DNA immunoprecipitated with RUVBL1 and inhibited H4K16Ac in the promoter region of C-myc. Overexpression of RUVBL1 in cells transfected with pre-miR-370-3p showed a higher enrichment of RUVBL1 immunoprecipitated and restored the inhibited H4K16Ac (Fig. 7d). Similarly, inhibition of miR-370-3p increased the presence of RUVBL1 and H4K16Ac at the indicated region and transfection of shRUVBL1 in miR-370-3p significantly inhibited H4K16Ac consistent with the decrease of RUVBL1 immunoprecipitated (Fig. 7e). These results showed that miR-370-3p inhibits H4K16Ac at the promoter region of C-myc partially via the downregulation of RUVBL1.

Next, we sought to find whether circMYO10 was involved in the H4K16Ac at the indicated region since circMYO10 was shown to activate Wnt/β-catenin signaling via miR-370-3p/RUVBL1 axis. We transfected MG63 cells with vector or shcircMYO10 or shcircMYO10 and miR-370-3p sponge or shcircMYO10 and RUVBL1 to construct stable cell lines. Consistent with our hypothesis, knockdown of circMYO10 inhibited the presence of RUVBL1 at the promoter region of C-myc and inhibited H4K16Ac (Fig. 7f). Furthermore, co-transfection with either RUVBL1 or miR-370-3p sponge partially abrogated the function of circMYO10 on H4K16Ac and the amount of RUVBL1 immunoprecipitated (Fig. 7f). Taken together, these results showed that miR-370-3p/RUVBL1 axis mediated the function of circMYO10 on H4K16Ac at the promoter region of C-myc.

### CircMYO10 acts as a sponge of miR-370-3p to promote tumorigenesis and metastasis in vivo

**Fig. 8:**
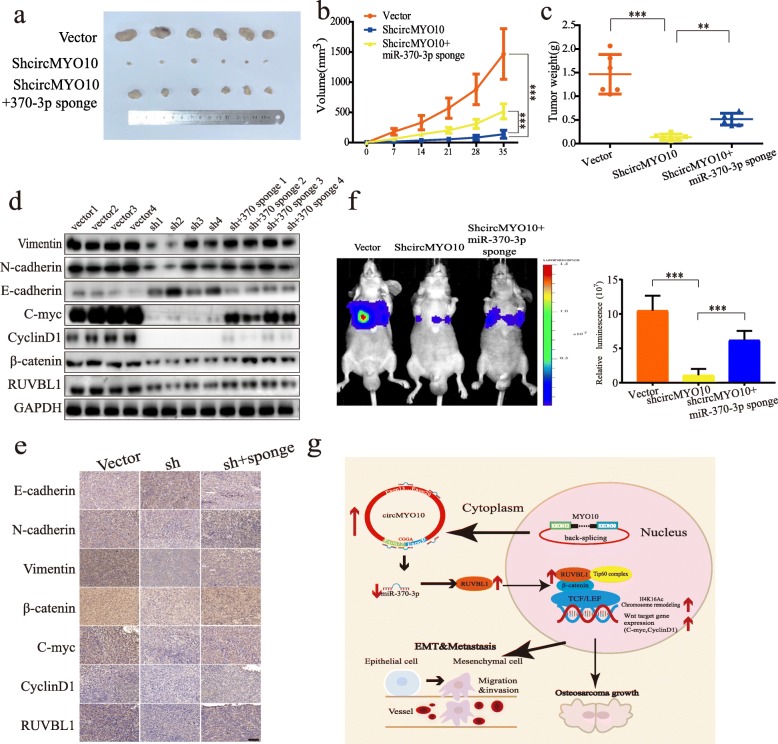
CircMYO10 acts as a miR-370-3p sponge to promote the growth and metastasis of OS cells in vivo. **a** Nude mice were injected subcutaneously with 10^7^ stable MG63 control cells or cells transfected with shcircMYO10 or cells co-transfected with circMYO10 shRNA and miR-370-3p sponge. Tumors were dissected, photographed, and weighed after 5 weeks. **b** Tumor volume was measured every week. Data represents the mean ± SD (*n* = 6). **c** Average tumor weight in each group at the end of the experiment (day 35). Data represents the mean ± SD (*n* = 6). **d** Western blot analysis of RUVBL1, β-catenin, C-myc, CyclinD1, Vimentin, E-cadherin, and N-cadherin of protein extracted from tumors. Four tumors were selected from each group for western blot analysis. Sh: shcircMYO10. Sh + sponge: shcircMYO10 + miR-370-3p sponge. **e** Immunohistochemistry analysis of RUVBL1, β-catenin, C-myc, CyclinD1, N-cadherin, Vimentin, and E-cadherin in tumors. Scale bars = 100 μm. **f** Representative images of Bioluminescence imaging of in vivo metastatic activity in nude mice 4 weeks after vail vein injection. **g** Model patterns of circMYO10/miR-370-3p/RUVBL1 axis. Three independent assays were performed in the above assays. **b**-**c**, **f** * *P* < 0.05, ** *P* < 0.01, *** *P* < 0.001 (Student’s t-test)

To explore whether circMYO10 and miR-370-3p are involved in tumorigenesis in vivo, we constructed control, circMYO10-inhibited, as well as circMYO10 and miR-370-3p double-inhibited stable cells, and subcutaneously injected stable cells of each group into nude mice. As shown in Fig. 8a-b, circMYO10 knockdown significantly decreased the tumor growth rate in vivo compared to that in the NC group, whereas double-inhibited cells demonstrated a higher growth rate than the circMYO10-inhibited group. Average wet weight of tumors in the circMYO10-inhibited group was also remarkably smaller than those in the vector group, and miR-370-3p inhibition attenuated the difference induced by circMYO10 knockdown (Fig. 8c). We then investigated the in vivo correlation of circMYO10, miR-370-3p, RUVBL1, β-catenin, C-myc, CyclinD1, and EMT markers. Consistent with the in vitro observations, the circMYO10-inhibited group displayed downregulated protein levels of RUVBL1, β-catenin, C-myc, Cyclin D1, N-cadherin, and Vimentin, with E-cadherin upregulated when compared to vector group. Inhibition of miR-370-3p partially abrogated this difference (Fig. 8d). A similar pattern was observed for RUVBL1, β-catenin, C-myc, Cyclin D1, and EMT markers by our immunohistochemistry analysis (Fig. 8e). Importantly, RUVBL1 was found to localize to both the cytoplasm and nucleus. As circMYO10 and miR-370-3p were shown to be associated with EMT in vivo, we wondered whether they were involved in the metastasis of OS cells. MG63 cells stably expressing luciferase and shcircMYO10 with or without the miR-370-3p sponge were injected via tail vein into 6-week-old nude mice. Bioluminescence imaging revealed that circMYO10 inhibition strongly inhibited the lung metastasis of OS cells compared with the control group and miR-370-3p inhibition in the circMYO10-inhibited group partially reversed this effect (Fig. 8f). Collectively, results showed that circMYO10 acts as a sponge for miR-370-3p in OS, both of which are inhibited to mediate the tumor-promoting effect of circMYO10 in vivo (Fig. 8g).

### MiR-877-3p mediates the process where circMYO10 regulates Wnt/β-catenin signaling

Besides miR-370-3p, our pull-down assays, dual luciferase assays, and FISH assays also showed that miR-877-3p binds to circMYO10 and circMYO10 contains three 8mer-1a sites for miR-877-3p (Fig. 3e-f and Additional file 9: Figure S8a). Interestingly, similar to miR-370-3p, miR-877-3p was downregulated in OS tissues and cell lines when compared to chondroma tissues and hFOB1.19 cell line (Additional file 10: Figure S9a-c) and it was shown to compromise the proliferation, migration, invasion and EMT ability of MG63 and U2OS cells as evidenced by our CCK-8 assays, colony formation assays, transwell assays and western blot analysis (Additional file 10:Figure S9d-h). Pathway enrichment analysis by Diana tools [59] showed that miR-877-3p may directly target LEF1 to inhibit Wnt/β-catenin signaling. Our dual-luciferase assays, western blot analysis and qRT-PCR analysis showed that miR-877-3p targets LEF1 directly and downregulates the expression of LEF1, β-catenin (protein level), C-myc and CyclinD1 which indicated that miR-877-3p inhibits Wnt/β-catenin signaling but further rescue experiment is needed to prove the involvement of LEF1 in miR-877-3p-mediated inhibition of Wnt/β-catenin signaling (Additional file 11: Figure S10a-e). Moreover, we showed that inhibition of miR-877-3p in circMYO10-inhibited cells rescued the inhibited Wnt/β-catenin signaling, compromised proliferation, migration and invasion ability in vitro as well as suppressed proliferation and EMT process in vivo (Additional file 12: Figure S11a-d and Additional file 13: Figure S12a-e). Those results indicated that miR-877-3p mediates the process where circMYO10 regulates Wnt/β-catenin signaling and further research is needed to prove the involvement of LEF1.

## Discussion

The 5-year survival rate of patients with OS has increased in the past 30 years. However, drug-resistant or metastatic OS remains refractory to treatment [4]. New treatments that are effective for refractory OS are needed.

Emerging evidence indicates that circRNAs are dysregulated in multiple cancers and may be involved in miRNA inhibition, EMT, and tumorigenesis, and thus could be potential therapeutic targets for OS [60, 61]. A previous study revealed that CircFAT1 acts as an miR-375 sponge to upregulate YAP1 and promote migration, invasion, and apoptosis evasion of OS cells [24]. Here, we showed that circMYO10 is significantly upregulated in OS tissues and cell lines. CircMYO10 knockdown inhibits OS cells growth, EMT, and metastasis in vitro and in vivo, indicating an oncogenic role of circMYO10 in OS and its potential value as a biomarker to predict the prognosis of patients with OS. Although it is not clear how circRNAs regulate the progression of cancer, emerging evidence suggests that they may function as miRNA sponges, undergo translation, or interact with transactivation elements to regulate the transcription of their parental gene [19, 62, 63]. Moreover, circRNAs, which are predominantly located in the cytoplasm, frequently act as miRNA sponges [64]. Bioinformatics analysis revealed that the 2867-bp circMYO10 is able to accommodate multiple potential binding sites for a multitude of miRNAs. Our pull-down, FISH and dual luciferase reporter assays revealed the direct binding of miR-370-3p to circMYO10. Moreover, we also found that circMYO10 promotes osteosarcoma progression by acting as a sponge for miR-370-3p to inhibit its anti-tumor effect, and upregulates the expression of its target gene, RUVBL1, which promotes histone H4 acetylation at the promoter region of C-myc. In addition, we also found miR-877-3p may also mediate the process where circMYO10 promotes Wnt/β-catenin signaling, and further research is needed.

Widely reported as a tumor suppressor, miR-370-3p is reported to inhibit Wnt signaling, which is recently clarified as a crucial driver of carcinoma metastasis [65], in thyroid cancer, bladder cancer, and glioma [47, 49, 50]. Importantly, miR-370-3p inhibits EMT process of glioma and bladder cancer cells in vitro [47, 49]. In this study, we found that miR-370-3p targets RUVBL1 directly to inhibit the interaction between RUVBL1 and β-catenin/LEF1 complex, which further suppresses H4K16 acetylation at the promoter region of C-myc and its transcription via effect on chromatin remodeling.

Widely reported as an oncogene in cancer, RUVBL1 is related to prognosis of patients with carcinoma [66, 67]. Depletion of RUVBL1 in renal cell carcinoma significantly decreased nuclear β-catenin expression and it was hypothesized that RUVBL1 may promote the relocation of β-catenin from the cadherins junctions to the nucleus [68]. Given that RUVBL1 is actually a coactivator of Wnt/β-catenin signaling [39, 40] and we showed that decreased nuclear β-catenin is accompanied with downregulated expression of Wnt target genes which is consistent with previous literature [40], the reduced amount of nuclear β-catenin may be due to the decreased β-catenin caused by inhibited Wnt/β-catenin signaling. However, though Wnt/β-catenin signaling positively regulates EMT process, there may be a different role of RUVBL1 in EMT since high expression of cytoplasmic RUVBL1 was correlated with poor prognosis and metastatic progression of patients with carcinoma which cannot be explained by augmented Wnt/β-catenin signaling by nuclear RUVBL1 [66]. In our study, we first identified the oncogenic role of RUVBL1 in OS and found that downregulation of RUVBL1 in OS cells inhibited cell proliferation and EMT process accompanied by inhibited Wnt/β-catenin signaling. In addition, cytoplasmic and nuclear localization of RUVBL1 was detected in both MG63 and U2OS cells. Further research is needed to clarify the role of cytoplasmic RUVBL1.

It was reported that RUVBL1 promotes the transcription activity of β-catenin via its effect on histone H4 acetylation [39, 40, 42]. Later study showed that RUVBL1 may not be necessary for the histone H3/4 acetylation in the regulation of KAI1, a tumor suppressor gene, since knockdown of RUVBL1 showed limited effect on histone H3/4 acetylation, but TIP60 is indispensable while knockdown of TIP60 significantly inhibited H3/4 acetylation [41]. However, coimmunoprecipitating acetylated histone H3 and H4 together in RUVBL1-inhibited cells may not be appropriate [41], since this may produce a different result if RUVBL1 is only involved in acetylation of specific sites in histone H4 while TIP60 is actually necessary for the acetylation of both histone H3 and H4. Supporting this, in the process of DNA repair, knockdown of RUVBL1 in Hela cells inhibited H4K16Ac and H4K12Ac while no difference is found in H2K5Ac and H4K5Ac, and methylated RUVBL1 promotes H4K16Ac to enhance the activity of TIP60 [56], all of which may indicate that the role of RUVBL1 in histone acetylation is site-specific. Moreover, we showed that RUVBL1 promotes H4K16Ac at the promoter region of C-myc to enhance the transcription activity of β-catenin/LEF1 complex. Knockdown of β-catenin inhibited H4K16Ac and recruitment of RUVBL1 to the promoter region of C-myc while TIP60 suppression significantly abrogated the enhanced H4K16Ac induced by RUVBL1 overexpression, showing little effect on the recruitment of RUVBL1. Since RUVBL1 is a subunit of TIP60 acetyltransferase complex [69, 70], these results may suggest that β-catenin recruits TIP60 histone acetyltransferase complex via RUVBL1 at this process (Fig. 8g), while the direct interaction between β-catenin and RUVBL1 is widely reported [39, 40, 42]. Since β-catenin plays a central role in canonical Wnt signaling, the presence of RUVBL1 in the regulation of Wnt target genes may be universal. Similar effect of RUVBL1 in C-myc-mediated transcription was also reported at the presence of TIP60 [71, 72].

Previously, there is no report about the role of circRNAs in histone acetylation and our study provided a new insight into the role of circRNAs in cancer in a way of chromatin remodeling. However, for this study, questions remain for that circMYO10 actually induced a stronger effect than miR-370-3p, indicating there may be other potential mechanisms for the role of circMYO10 in OS and further research is needed.

## Conclusions

We demonstrated that circMYO10 is upregulated in OS tissues and cell lines. CircMYO10 activates Wnt/β-catenin signaling by regulating miR-370-3p/RUVBL1 axis to promote H4K16Ac at the promoter region of β-catenin/LEF1 target genes. The data provides a link between circRNAs, Wnt/ β-catenin signaling, chromatin remodeling and OS progression.

## Supplementary information

**Additional file 1.** Supplementary materials and methods.

**Additional file 2: Figure S1.** The expression of either circMYO10 or miR-370-3p detected by qRT-PCR. (a) Expression of circMYO10 upon transfection with Si-circMYO10. (b) Efficiency of miR-370-3p mimics and inhibitors were measured in both MG63 and U2OS cells. (a-b) Data represents the mean ± SD (*n* = 3). Three independent assays were performed in the above assays. * *P* < 0.05, ** *P* < 0.01, *** *P* < 0.001 (Student’s t-test).

**Additional file 3: Figure S2.** The seed regions between miR-877-3p and circMYO10. (a) Schematic illustration showing complementarity to the miR-370-3p sequence in circMYO10. (TIF 1399 kb)

**Additional file 4: Figure S3.** MiR-370-3p showed a strong effect on the migration, invasion, and EMT process than miR-877-3p in MG63 and U2OS cells. (a) MiR-370-3p and miR-877-3p inhibited MG63 and U2OS cells to migrate and invade through transwells. Scale bars = 50 μ m. Data represents the mean ± SD. (b) Western blot analysis of Vimentin, N-cadherin and E-cadherin in cells transfected with either miR-370-3p or miR-877-3p. Three independent assays were performed in the above assays. (a) * *P* < 0.05, ** *P* < 0.01, *** *P* < 0.001 (Student’s t-test).

**Additional file 5: Figure S4.** RUVBL1 is an oncogene in osteosarcoma. (a) CCK-8 assays for MG63 and U2OS cells transfected with either NC or RUVBL1 SiRNA. Data represents the mean ± SD (*n* = 18) Data represents the mean ± SD. (b) Anchorage -independent colony formation of MG63 and U2OS cells was inhibited upon RUVBL1 knockdown. Scale bars = 50 μ m. (c) RUVBL1 inhibited compromised the migration ability of MG63 and U2OS cells. Scale bars = 200 μ m. Data represents the mean ± SD (*n* = 3) Data represents the mean ± SD. Three independent assays were performed in the above assays. (a, c) * *P* < 0.05, ** *P* < 0.01, *** *P* < 0.001 (Student’s t-test).

**Additional file 6: Figure S5.** RUVBL1 positively regulates Wnt/β-catenin signaling. (a) Downregulation of C-myc, cyclinD1 and β-catenin was detected in RUVBL1-inhibited MG63 and U2OS cells by (a) qRT-PCR and (b) immunofluorescence analysis. (a) Data represents the mean ± SD. (b) Scale bars = 50 μ m. Three independent assays were performed in the above assays. (a-b) * *P* < 0.05, ** *P* < 0.01, *** *P* < 0.001 (Student’s t-test).

**Additional file 7: Figure S6.** MiR-370-3p inhibits Wnt/β-catenin signaling. (a-c) Transfection with miR-370-3p mimics significantly downregulated the expression of β-catenin, C-myc and cyclinD1 as revealed by (a) qRT-PCR, (b) western blot analysis and (c) immunofluorescence analysis, while inhibition of miR-370-3p showed a contrary result. (a) Data represents the mean ± SD. (c) Scale bars = 50 μ m. Three independent assays were performed in the above assays. (a) * *P* < 0.05, ** *P* < 0.01, *** *P* < 0.001 (Student’s t-test).

**Additional file 8: Figure S7.** Either overexpression of RUVBL1 or inhibition of miR-370-3p partially restored the phenotypes caused by the circMYO10 knockdown. (a-c) Transfection with either miR-370-3p sponge or RUVBL1 partially abrogated the inhibited proliferation ability of MG63 and U2OS cells induced by shcircMYO10. (a) CCK-8 analysis, (b) colony formation assays, (c) cell cycle analysis. (a) Data represents the mean ± SD (*n* = 18). (b-c) Data represents the mean ± SD (*n* = 3). Three independent assays were performed in the above assays. (a-b) * *P* < 0.05, ** *P* < 0.01, *** *P* < 0.001 (Student’s t-test).

**Additional file 9: Figure S8.** High degree colocalization of miR-877-3p and circMYO10 was detected. (a) FISH assays showed high degree colocalization of circMYO10 and miR-877-3p.

**Additional file 10: Figure S9.** MiR-877-3p inhibits OS progression. (a) The expression of miR-877-3p in ten paired chondroma (*n* = 10) and osteosarcoma tissues (*n* = 10) was measured by qRT-PCR and FISH assays. (a) Data represents the mean ± SD (*n* = 90 per group). (b) FISH assays showed miR-877-3p expression is lower in human OS tissue than in chondroma tissue. Representative images are shown. Scale bars = 100 μm. (c) The expression of miR-877-3p in hFOB1.19, 143B, U2OS, HOS, MG63, and SJSA-1 was measured by qRT-PCR. Data represents the mean ± SD (*n* = 9). (d) The efficiency of miR-877-3p mimics and miR-877-3p inhibitor was detected by qRT-PCR. Data represents the mean ± SD (*n* = 9). (e) Transwell migration and invasion assays revealed enhanced migration and invasion ability of MG63 and U2OS cells after transfection with miR-877-3p inhibitors. Scale bars = 50 μ m. Data represents the mean ± SD. (f-g) Proliferation ability of MG63 and U2OS cells transfected with either miR-877-3p mimics or miR-877-3p inhibitor was evaluated by CCK-8 assays and colony formation assays. Data represents the mean ± SD. (h) The changes of EMT markers, N-cadherin, E-cadherin, and Vimentin upon miR-877-3p overexpression and inhibition were detected by western blot analysis. Three independent assays were performed in the above assays. (a, c, d-g) * *P* < 0.05, ** *P* < 0.01, *** *P* < 0.001 (Student’s t-test).

**Additional file 11: Figure S10.** LEF1 is a direct target of miR-877-3p and miR-877-3p inhibits Wnt/β-catenin signaling. (a) Pathway enrichment analysis was conducted for miR-877-3p via mirPath v. 3. (b) Complementary sequences of the LEF1 3′ UTR for miR-877-3p. (c) 293 T cells were co-transfected with miR-877-3p and a luciferase reporter plasmid containing either wild type or mutated LEF1 3′ UTRs. Data represents the mean ± SD (*n* = 9). (d) The changes in levels of LEF1, β-catenin, C-myc, and CyclinD1 were detected by western blot. (e) Relative mRNA expression of LEF1, β-catenin, C-myc, and cyclinD1 was measured by qRT-PCR. Data represents the mean ± SD (*n* = 9). Three independent assays were performed in the above assays. (c, e) * *P* < 0.05, ** *P* < 0.01, *** *P* < 0.001 (Student’s t-test).

**Additional file 12: Figure S11.** MiR-877-3p is involved in the process where circMYO10 promotes osteosarcoma progression via activated Wnt/β-catenin signaling. (a) The expression of miR-877-3p in MG63 cells and U2OS cells stably transfected with shcircMYO10 or shcircMYO10 plus miR-877-3p sponge. Data represents the mean ± SD. (b) Migration and invasion assay in transwells showed that miR-877-3p inhibition promoted the migration and invasion ability of cells stably transfected with shcircMYO10 and miR-877-3p overexpression induced contrary results. Scale bars = 50 μm. Data represents the mean ± SD. (c-d) Inhibition of miR-877-3p partially abrogated the suppressive effect of shcircMYO10 on proliferation ability of both MG63 and U2OS cells. Data represent the mean ± SD. (e) The effect of miR-877-3p on EMT and Wnt/β-catenin signaling in cells stably transfected with shcircMYO10 was measured by western blot. Three independent assays were performed in the above assays. (a-d) * *P* < 0.05, ** *P* < 0.01, *** *P* < 0.001 (Student’s t-test).

**Additional file 13: Figure S12.** (a-c) Nude mice were injected subcutaneously with 10^7^ stable MG63 control cells, cells transfected with shcircMYO10, or cells co-transfected with circMYO10 shRNA and miR-877-3p sponge. Tumors were dissected, photographed, and weighed after 5 weeks. (b) Tumor volume was measured every week. Data represent the mean ± SD (*n* = 5). (c) Average wet tumor weight in each group at the end of the experiment (day 35). Data represents the mean ± SD (*n* = 5). (d) Western blot analysis of LEF1, β-catenin, C-myc, CyclinD1, Vimentin, E-cadherin, and N-cadherin of protein extracted from tumors. Sh: shcircMYO10. Sh + sponge: shcircMYO10 + miR-877-3p sponge. (e) Immunohistochemistry analysis of LEF1, β-catenin, C-myc, CyclinD1, Vimentin, N-cadherin, and E-cadherin in tumors. Scale bars = 100 μm. Three independent assays were performed in the above assays. (c-d) * *P* < 0.05, ** *P* < 0.01, *** *P* < 0.001 (Student’s t-test).

## Data Availability

The datasets used and/or analyzed during the current study are available from the corresponding author on reasonable request.

## Abbreviations

3′-UTR: 3′-untranslated region
APC: Adenomatosis polyposis coli
circRNA: circular RNA
EMT: Epithelial–mesenchymal transition
FISH: Fluorescent In Situ Hybridization
H4K16Ac: Histone H4K16 acetylation
lncRNA: Long non-coding RNA
LUC: Firefly luciferase
miRNA: microRNA
OS: Osteosarcoma
qPCR: Quantitative real-time PCR
qRT-PCR: Quantitative reverse transcription PCR
RLUC: Renilla luciferase
