# Correction to: CircMYO10 promotes osteosarcoma progression by regulating miR-370-3p/RUVBL1 axis to enhance the transcriptional activity of β-catenin/LEF1 complex via effects on chromatin remodeling

**DOI:** 10.1186/s12943-020-01193-7

**Published:** 2020-04-14

**Authors:** Junxin Chen, Gang Liu, Yizheng Wu, Jianjun Ma, Hongfei Wu, Ziang Xie, Shuai Chen, Yute Yang, Shengyu Wang, Panyang Shen, Yifan Fang, Shunwu Fan, Shuying Shen, Xiangqian Fang

**Affiliations:** 1 Department of Orthopaedic Surgery, Sir Run Run Shaw Hospital, Medical College of Zhejiang University & Key Laboratory of Musculoskeletal System Degeneration and Regeneration Translational Research of Zhejiang Province, 3 East Qingchun Road, Hangzhou, 310016 Zhejiang Province China; 2 Department of Spinal Surgery, Orthopaedic Medical Center, Hospital of Zhejiang Armed Police Corps, Jiaxing, Zhejiang Province China; 3 Hangzhou Foreign Language School, Hangzhou, Zhejiang Province China

**Correction to: Mol Cancer (2019) 18:150**

**https://doi.org/10.1186/s12943-019-1076-1**

Following the publication of the original paper [1], one corresponding author was inadvertently missed. Prof. Shunwu Fan provided many important suggestions/contribution to this research and made effort to attain success for this study. Hence, the authors decided and agreed that Prof. Shunwu Fan should be listed as one of the corresponding authors. Authorgroup section above has been updated.
