# Supplementary material for: CircMYO10 promotes osteosarcoma progression by regulating miR-370-3p/RUVBL1 axis to enhance the transcriptional activity of β-catenin/LEF1 complex via effects on chromatin remodeling
Source: Mol Cancer. 2019 Oct 29;18:150. doi: 10.1186/s12943-019-1076-1 (PMC6819556; doi:10.1186/s12943-019-1076-1)
Supplement: Supplementary file 1 — Additional file 1. Supplementary materials and methods. [file 12943_2019_1076_MOESM1_ESM.docx]

**Supplementary Materials and Methods**

***Antibodies, mice and other materials***

##### Anti-E-cadherin (ab133597), Vimentin (ab92547), C-myc (ab32072), CyclinD1 (ab226977), RUVBL1 (ab51500) antibodies were obtained from Abcam. Anti-GAPDH (10494-1-AP), β-catenin (51067-2-AP), β-catenin (66379-1-lg), RUVBL1(10210-2-AP), N-cadherin (22018-1-AP), LaminB1 (12987-1-AP) antibodies were purchased from Proteintech. Anti-LEF1 (76010), TIP60 (12058), Acetylated H4K16 (13534) antibodies were obtained from Cell signaling Technology. Alexa Fluor 488- and Alexa Fluor 594- conjugated secondary antibodies were obtained from Life Technologies. MiR-370-3p and miR-877-3p sponge was made by cloning a stretch of three perfect match miR-370-3p and miR-877-3p sponge antisense oligomers into the LV3 (H1/GFP&Puro) lentiviral vector relatively. SiRNA oligonucleotides toward circMYO10, RUVBL1, and control siRNA were purchased from RiboBio. MiR-370-3p and miR-877-3p mimics/ inhibitors were obtained from GenePharma. Common chemicals were from Sigma or Sangon Biotech.

Real-time PCR
Total RNA was extracted using TRIzol reagent and Ultrapure RNA Kit (CWBio). Reverse transcription was performed using a HiFiScript cDNA Synthesis Kit (CWBio), and cDNA amplification was performed using SYBR Green Mixture (CWBio). The miRNA levels were detected using miRNA purification, miRNA cDNA synthesis, and miRNA real-time PCR (qPCR) assay kits (all from CWBio) according to the manufacturer’s instructions. Details of primers are provided in Supplementary Materials.

Western blotting
Protein was extracted from samples with RIPA Lysis Buffer (CWBio, Beijing, China). Cell extracts were boiled in loading buffer and equal quantities of cell extracts were separated using 10% sodium dodecyl sulfate-polyacrylamide gel electrophoresis (SDS-PAGE) and transferred to polyvinylidene fluoride membranes (Merck KGaA, Darmstadt, Germany). The membranes were blocked with 5% nonfat milk at 25°C and then incubated with primary antibody, followed by incubation in the presence of secondary antibody. Tris-buffered saline Tween was used to wash the membrane following exposure to primary antibody or secondary body three times, with 10 min for each wash. Proteins were detected using an enhanced chemiluminescence substrate kit (Thermo Fisher Scientific, Waltham, MA, USA) and analyzed using ImageJ software. The expression of glyceraldehyde-3-phosphate dehydrogenase (GAPDH) was used as a loading control. Detailed information of antibodies is provided in the Supplementary Materials.

Viral infection

Lentiviral infection was used to produce stably expressing cells. Packaging plasmids (pSPAX2 and pMD2G) were co-transfected with viral vectors into HEK293T cells. 48h later, the culture medium was filtered through a 0.45 μm filter, supplemented with 5 μg/ml polybrene, and used for infection of target cells. After another 48 h, 1 μg/ml puromycin was used to select the infected cells.

Transfection

Small interfering RNAs (siRNAs) and miRNA mimics were purchased from GenePharma (Shanghai, China). Lipofectamine IMAX (Life Technologies, Carlsbad, CA, USA) was used for transfection of siRNA, miRNA mimics, and miRNA inhibitors. Lipofectamine 3000 (Life Technologies) was used for transfection of plasmids. Transfection was carried out according to the manufacturer’s instructions.

Primers used in this study are offered as follows:

| Human GAPDH Forward | AGCCACATCGCTCAGACAC |
| --- | --- |
| Human GAPDH Reverse | GCCCAATACGACCAAATCC |
| Human CircMYO10 Forward | CAGCCCTGATAGGCAGTAGAC |
| Human CircMYO10 Reverse | TCATTGCCTTTGATCCTGCTGT |
| Human MYO10 Forward | ACTTTTATGTGAAGCCCAGAGT |
| Human MYO10 Reverse | TCCTGGTTGTTGCGGCTT |
| Human LEF1 Forward | AGAACACCCCGATGACGGA |
| Human LEF1 Reverse | GGCATCATTATGTACCCGGAAT |
| Human U6 Forward | CTCGCTTCGGCAGCACA |
| Human U6 Reverse | AACGCTTCACGAATTTGCGT |
| Human RUVBL1 Forward | AGGTGAAGAGCACTACGAAGA |
| Human RUVBL1 Reverse | CTACTATGACGCCACATGCCT |
| Human CTNNB1 Forward | AAAATGGCAGTGCGTTTAG |
| Human CTNNB1 Reverse | TTTGAAGGCAGTCTGTCGTA |
| Human MYC Forward | CGTCCTCGGATTCTCTGCTC |
| Human MYC Reverse | GCTGCGTAGTTGTGCTGATG |
| Human CyclinD1 Forward | TGACCCCGCACGATTTCATT |
| Human CyclinD1 Reverse | CATGGAGGGCGGATTGGAAA |
| Human DFFA Forward | GCGGAAGGGGGTCGAGTA |
| Human DFFA Reverse | CCACTATGGTGCCATCCTCTG |
| Human AP1S1 Forward | CGGTTCATGCTATTATTCAGCCG |
| Human AP1S1 Reverse | CCGTTCCTTGTCCGAAGTGG |
| Human FADS1 Forward | CTACCCCGCGCTACTTCAC |
| Human FADS1 Reverse | CGGTCGATCACTAGCCACC |
| Human SNF8 Forward | AGCCCAGATGTCAAAGCAGTT |
| Human SNF8 Reverse | AGGCACACTTCGATAATTTGGAC |
