# Supplementary figures and images for: CircMYO10 promotes osteosarcoma progression by regulating miR-370-3p/RUVBL1 axis to enhance the transcriptional activity of β-catenin/LEF1 complex via effects on chromatin remodeling
Source: Mol Cancer. 2019 Oct 29;18:150. doi: 10.1186/s12943-019-1076-1 (PMC6819556; doi:10.1186/s12943-019-1076-1)

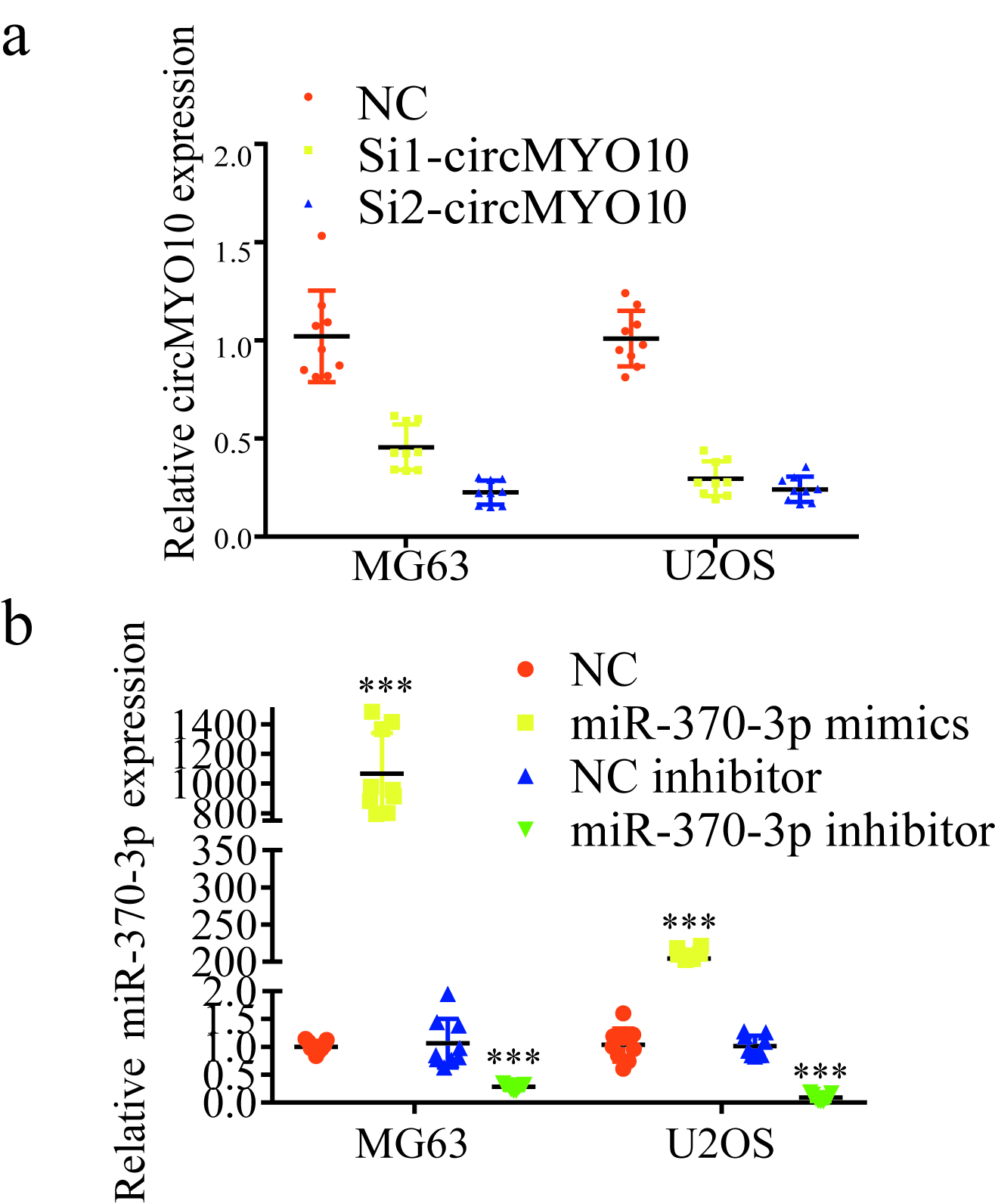

Supplement: Supplementary file 2 — Additional file 2: Figure S1. The expression of either circMYO10 or miR-370-3p detected by qRT-PCR. (a) Expression of circMYO10 upon transfection with Si-circMYO10. (b) Efficiency of miR-370-3p mimics and inhibitors were measured in both MG63 and U2OS cells. (a-b) Data represents the mean ± SD (n = 3). Three independent assays were performed in the above assays. * P < 0.05, ** P < 0.01, *** P < 0.001 (Student’s t-test). [file 12943_2019_1076_MOESM2_ESM.tif]

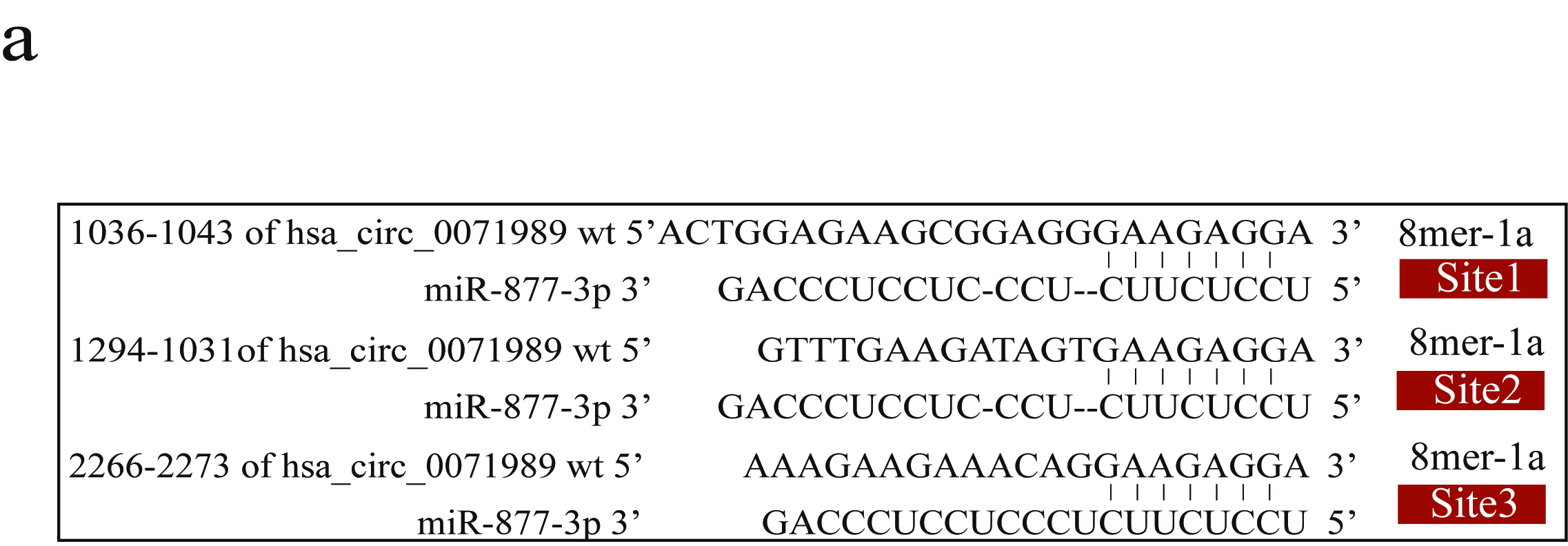

Supplement: Supplementary file 3 — Additional file 3: Figure S2. The seed regions between miR-877-3p and circMYO10. (a) Schematic illustration showing complementarity to the miR-370-3p sequence in circMYO10. (TIF 1399 kb) [file 12943_2019_1076_MOESM3_ESM.tif]

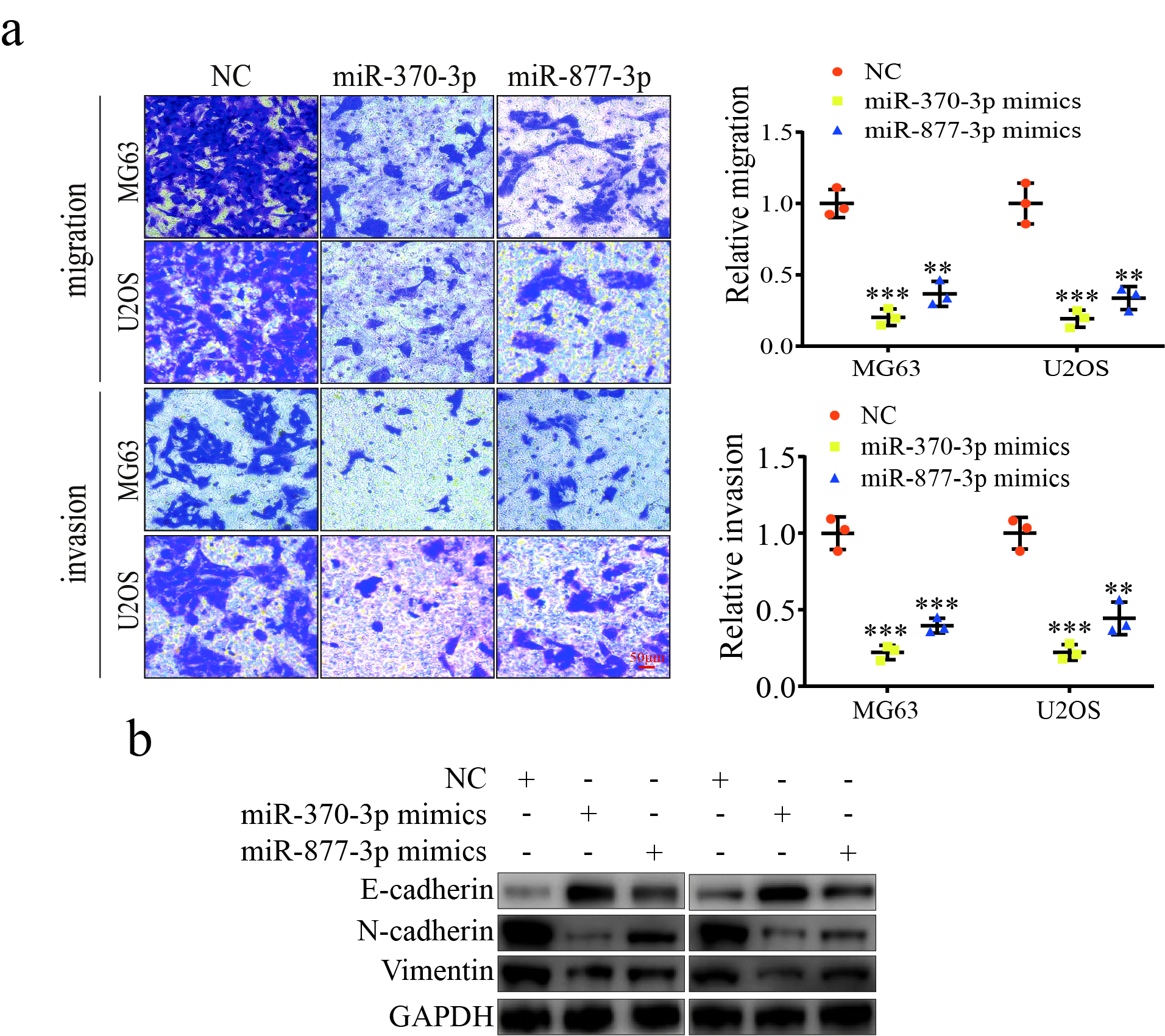

Supplement: Supplementary file 4 — Additional file 4: Figure S3. MiR-370-3p showed a strong effect on the migration, invasion, and EMT process than miR-877-3p in MG63 and U2OS cells. (a) MiR-370-3p and miR-877-3p inhibited MG63 and U2OS cells to migrate and invade through transwells. Scale bars = 50 μ m. Data represents the mean ± SD. (b) Western blot analysis of Vimentin, N-cadherin and E-cadherin in cells transfected with either miR-370-3p or miR-877-3p. Three independent assays were performed in the above assays. (a) * P < 0.05, ** P < 0.01, *** P < 0.001 (Student’s t-test). [file 12943_2019_1076_MOESM4_ESM.tif]

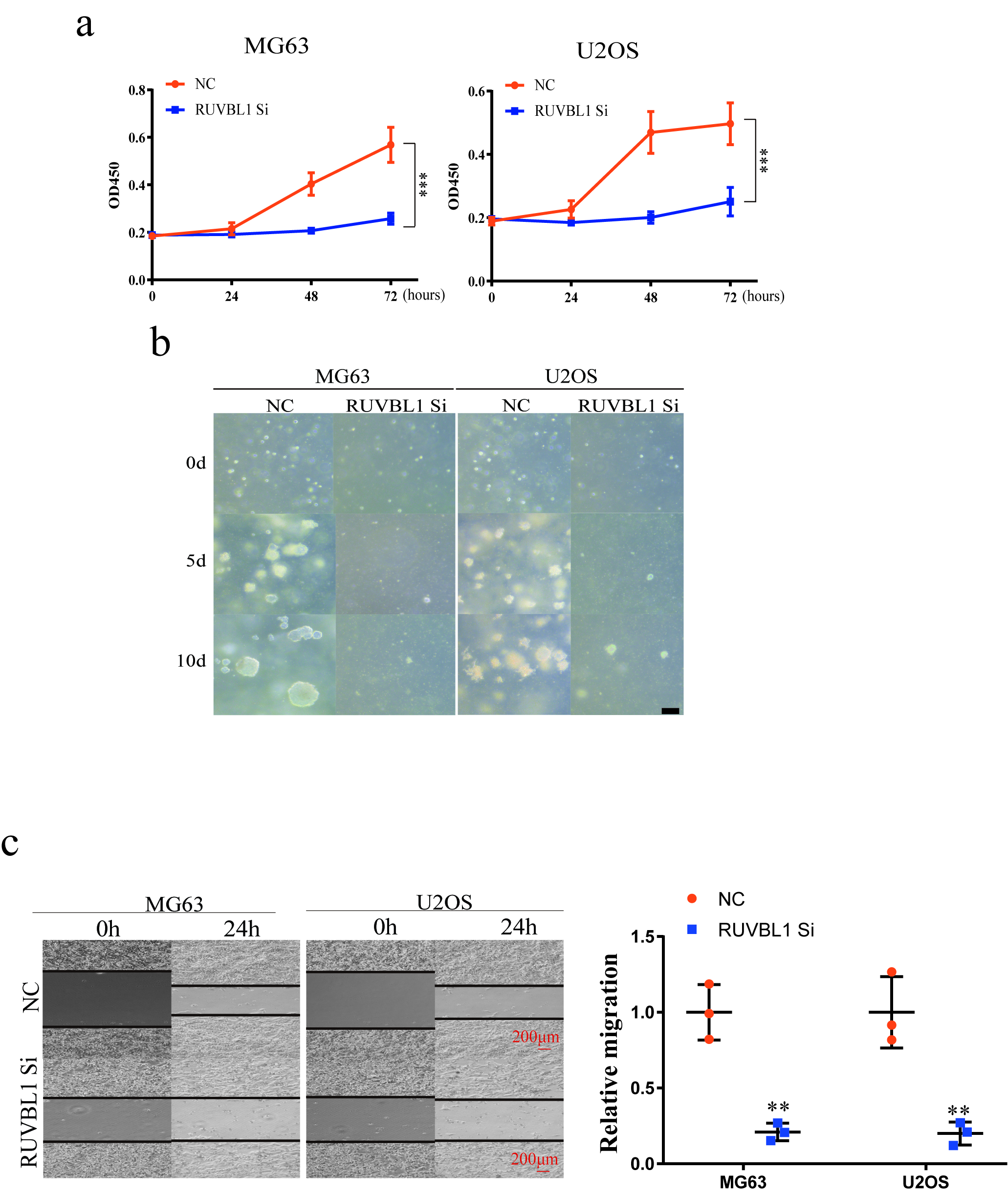

Supplement: Supplementary file 5 — Additional file 5: Figure S4. RUVBL1 is an oncogene in osteosarcoma. (a) CCK-8 assays for MG63 and U2OS cells transfected with either NC or RUVBL1 SiRNA. Data represents the mean ± SD (n = 18) Data represents the mean ± SD. (b) Anchorage -independent colony formation of MG63 and U2OS cells was inhibited upon RUVBL1 knockdown. Scale bars = 50 μ m. (c) RUVBL1 inhibited compromised the migration ability of MG63 and U2OS cells. Scale bars = 200 μ m. Data represents the mean ± SD (n = 3) Data represents the mean ± SD. Three independent assays were performed in the above assays. (a, c) * P < 0.05, ** P < 0.01, *** P < 0.001 (Student’s t-test). [file 12943_2019_1076_MOESM5_ESM.tif]

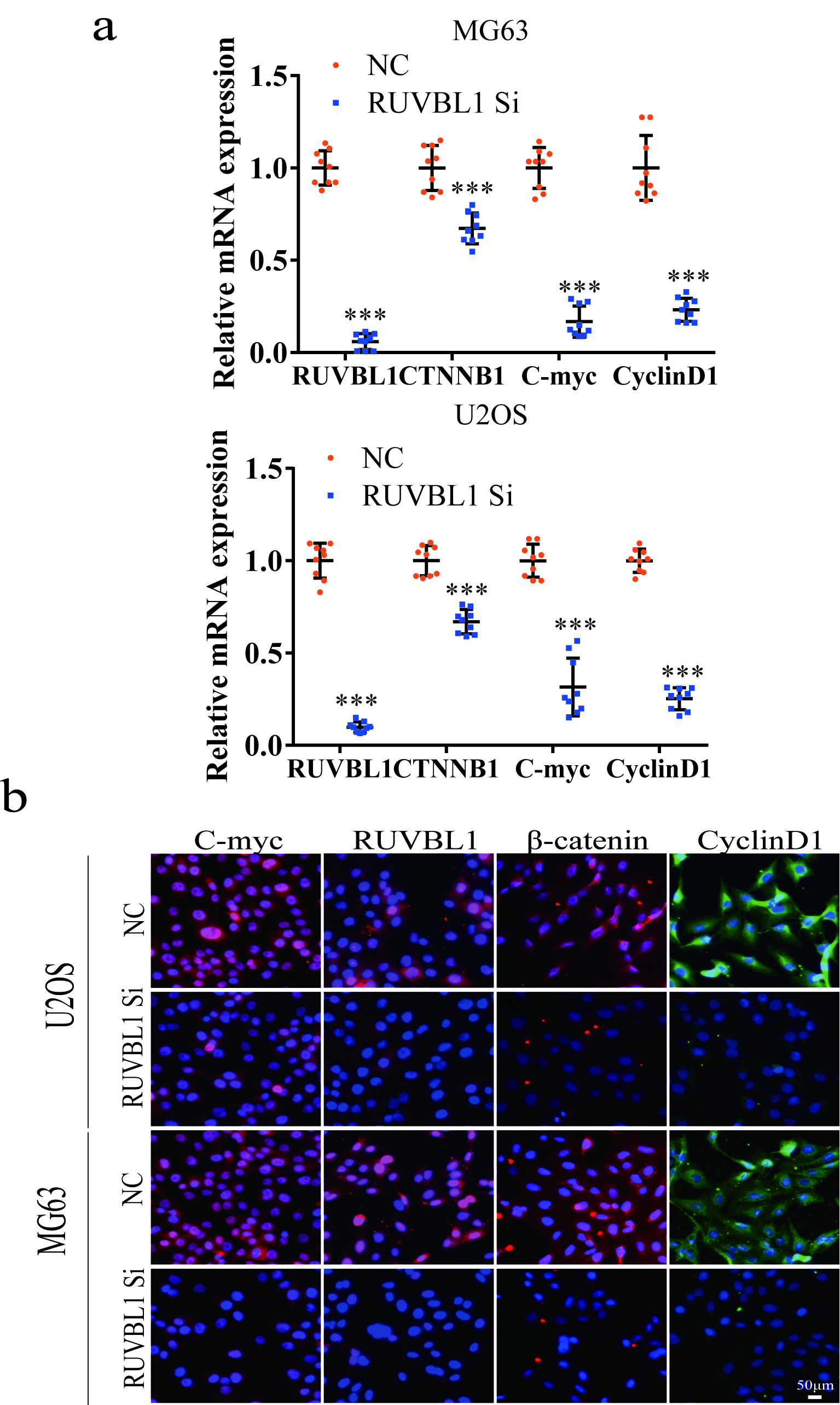

Supplement: Supplementary file 6 — Additional file 6: Figure S5. RUVBL1 positively regulates Wnt/β-catenin signaling. (a) Downregulation of C-myc, cyclinD1 and β-catenin was detected in RUVBL1-inhibited MG63 and U2OS cells by (a) qRT-PCR and (b) immunofluorescence analysis. (a) Data represents the mean ± SD. (b) Scale bars = 50 μ m. Three independent assays were performed in the above assays. (a-b) * P < 0.05, ** P < 0.01, *** P < 0.001 (Student’s t-test). [file 12943_2019_1076_MOESM6_ESM.tif]

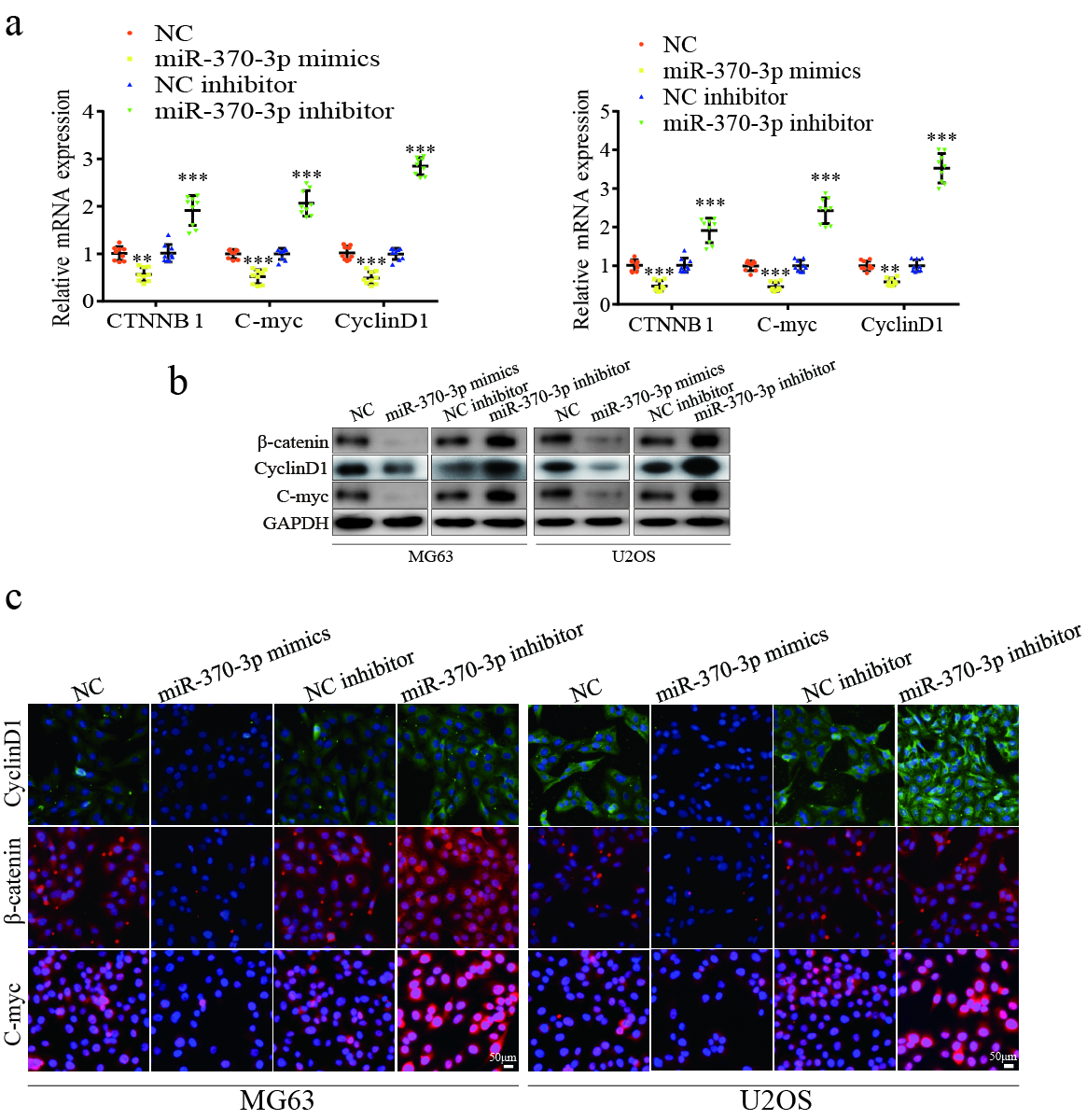

Supplement: Supplementary file 7 — Additional file 7: Figure S6. MiR-370-3p inhibits Wnt/β-catenin signaling. (a-c) Transfection with miR-370-3p mimics significantly downregulated the expression of β-catenin, C-myc and cyclinD1 as revealed by (a) qRT-PCR, (b) western blot analysis and (c) immunofluorescence analysis, while inhibition of miR-370-3p showed a contrary result. (a) Data represents the mean ± SD. (c) Scale bars = 50 μ m. Three independent assays were performed in the above assays. (a) * P < 0.05, ** P < 0.01, *** P < 0.001 (Student’s t-test). [file 12943_2019_1076_MOESM7_ESM.tif]

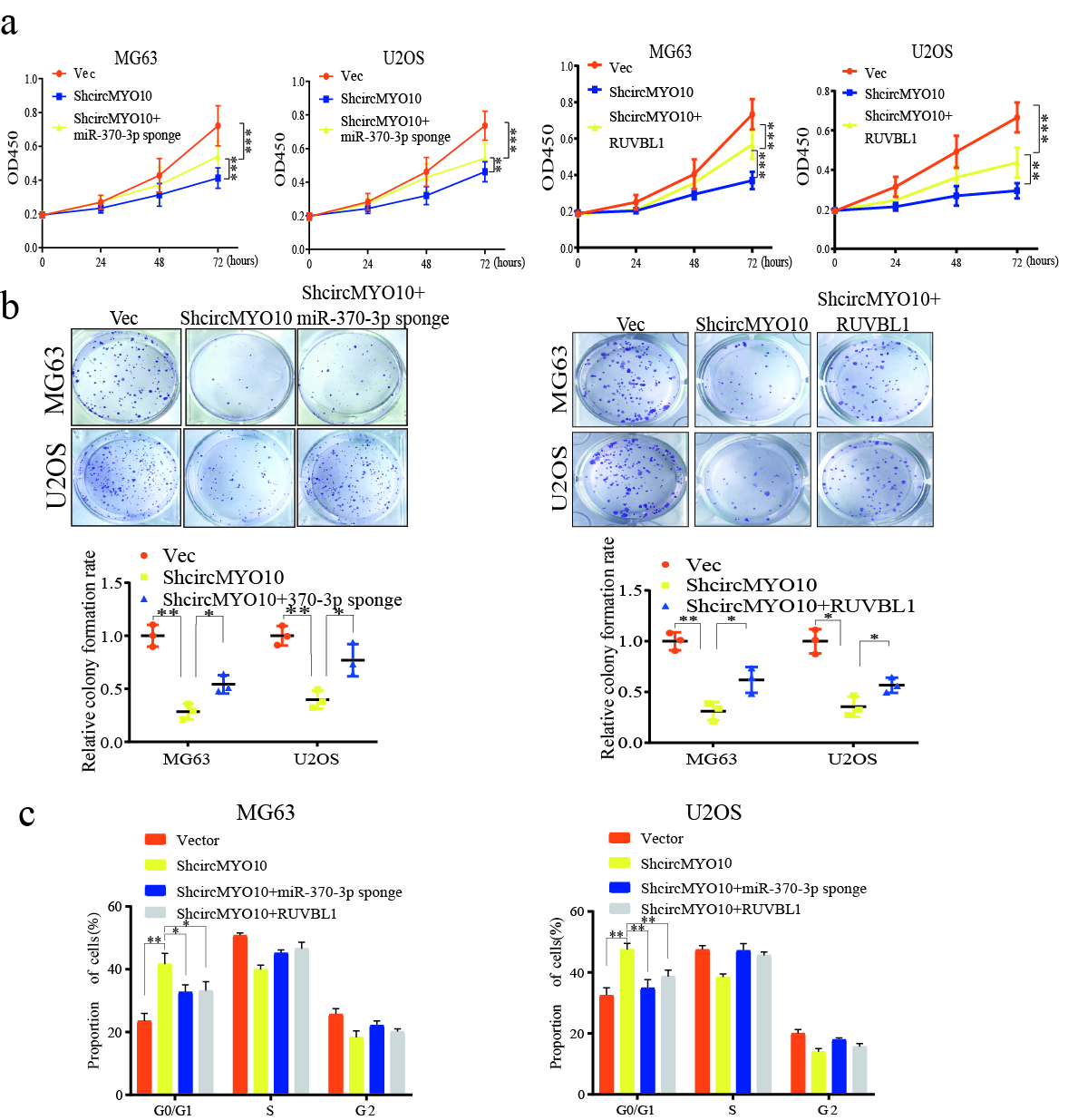

Supplement: Supplementary file 8 — Additional file 8: Figure S7. Either overexpression of RUVBL1 or inhibition of miR-370-3p partially restored the phenotypes caused by the circMYO10 knockdown. (a-c) Transfection with either miR-370-3p sponge or RUVBL1 partially abrogated the inhibited proliferation ability of MG63 and U2OS cells induced by shcircMYO10. (a) CCK-8 analysis, (b) colony formation assays, (c) cell cycle analysis. (a) Data represents the mean ± SD (n = 18). (b-c) Data represents the mean ± SD (n = 3). Three independent assays were performed in the above assays. (a-b) * P < 0.05, ** P < 0.01, *** P < 0.001 (Student’s t-test). [file 12943_2019_1076_MOESM8_ESM.tif]

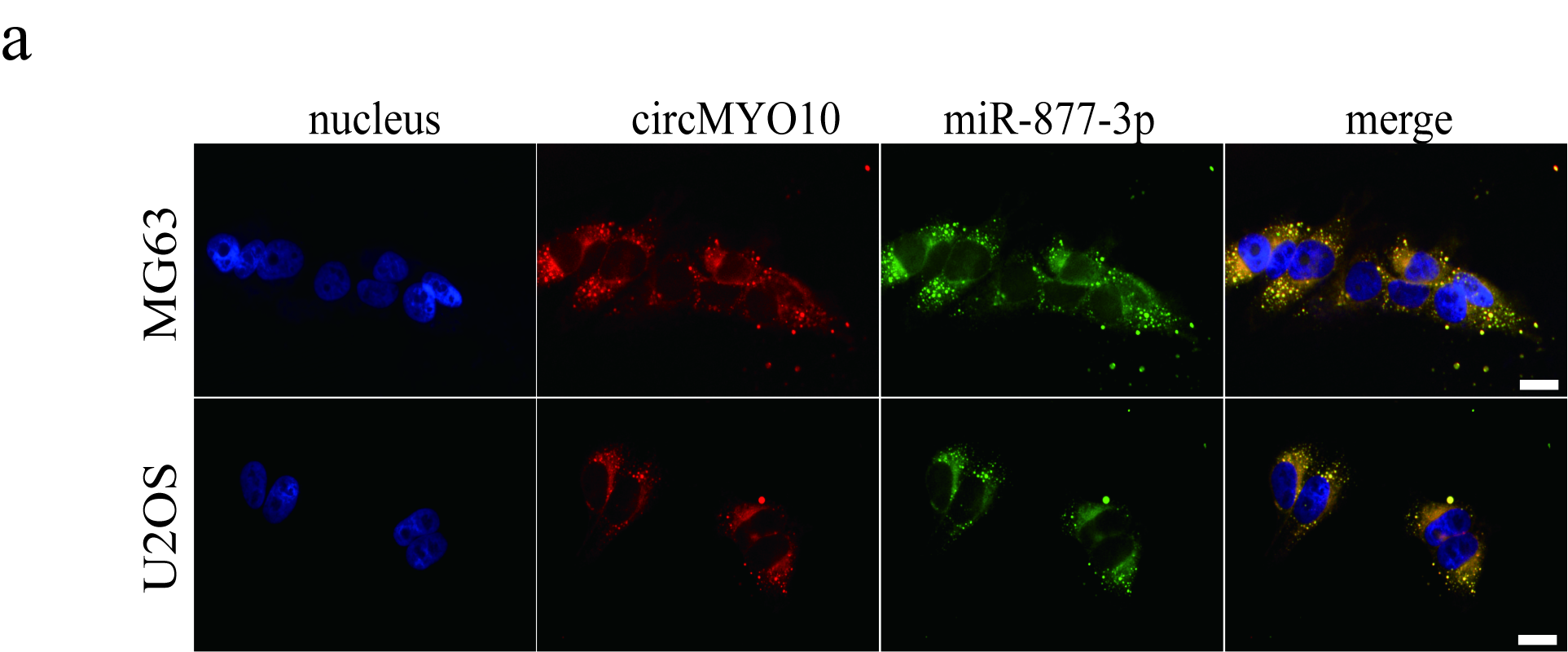

Supplement: Supplementary file 9 — Additional file 9: Figure S8. High degree colocalization of miR-877-3p and circMYO10 was detected. (a) FISH assays showed high degree colocalization of circMYO10 and miR-877-3p. [file 12943_2019_1076_MOESM9_ESM.tif]

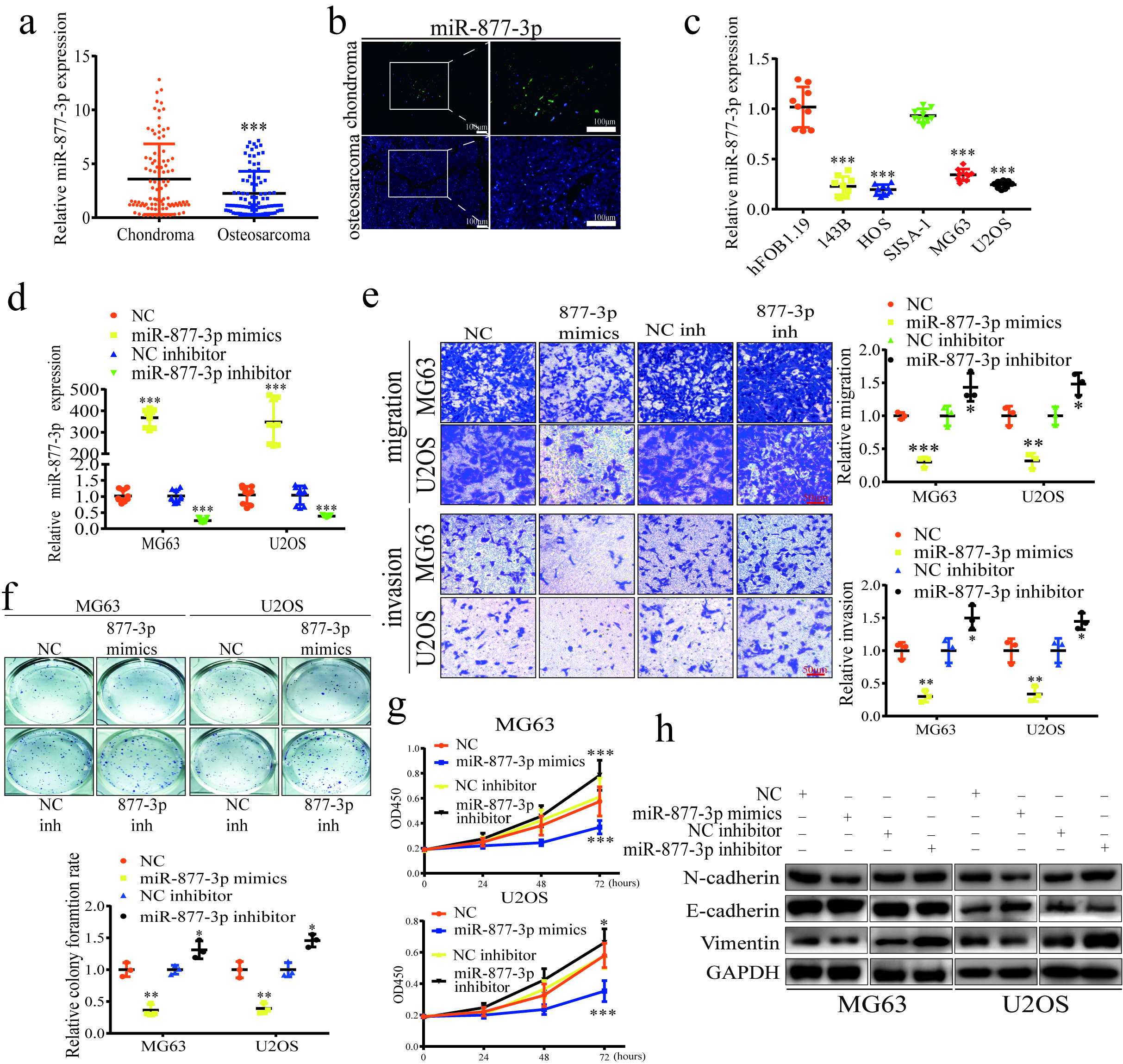

Supplement: Supplementary file 10 — Additional file 10: Figure S9. MiR-877-3p inhibits OS progression. (a) The expression of miR-877-3p in ten paired chondroma (n = 10) and osteosarcoma tissues (n = 10) was measured by qRT-PCR and FISH assays. (a) Data represents the mean ± SD (n = 90 per group). (b) FISH assays showed miR-877-3p expression is lower in human OS tissue than in chondroma tissue. Representative images are shown. Scale bars = 100 μm. (c) The expression of miR-877-3p in hFOB1.19, 143B, U2OS, HOS, MG63, and SJSA-1 was measured by qRT-PCR. Data represents the mean ± SD (n = 9). (d) The efficiency of miR-877-3p mimics and miR-877-3p inhibitor was detected by qRT-PCR. Data represents the mean ± SD (n = 9). (e) Transwell migration and invasion assays revealed enhanced migration and invasion ability of MG63 and U2OS cells after transfection with miR-877-3p inhibitors. Scale bars = 50 μ m. Data represents the mean ± SD. (f-g) Proliferation ability of MG63 and U2OS cells transfected with either miR-877-3p mimics or miR-877-3p inhibitor was evaluated by CCK-8 assays and colony formation assays. Data represents the mean ± SD. (h) The changes of EMT markers, N-cadherin, E-cadherin, and Vimentin upon miR-877-3p overexpression and inhibition were detected by western blot analysis. Three independent assays were performed in the above assays. (a, c, d-g) * P < 0.05, ** P < 0.01, *** P < 0.001 (Student’s t-test). [file 12943_2019_1076_MOESM10_ESM.tif]

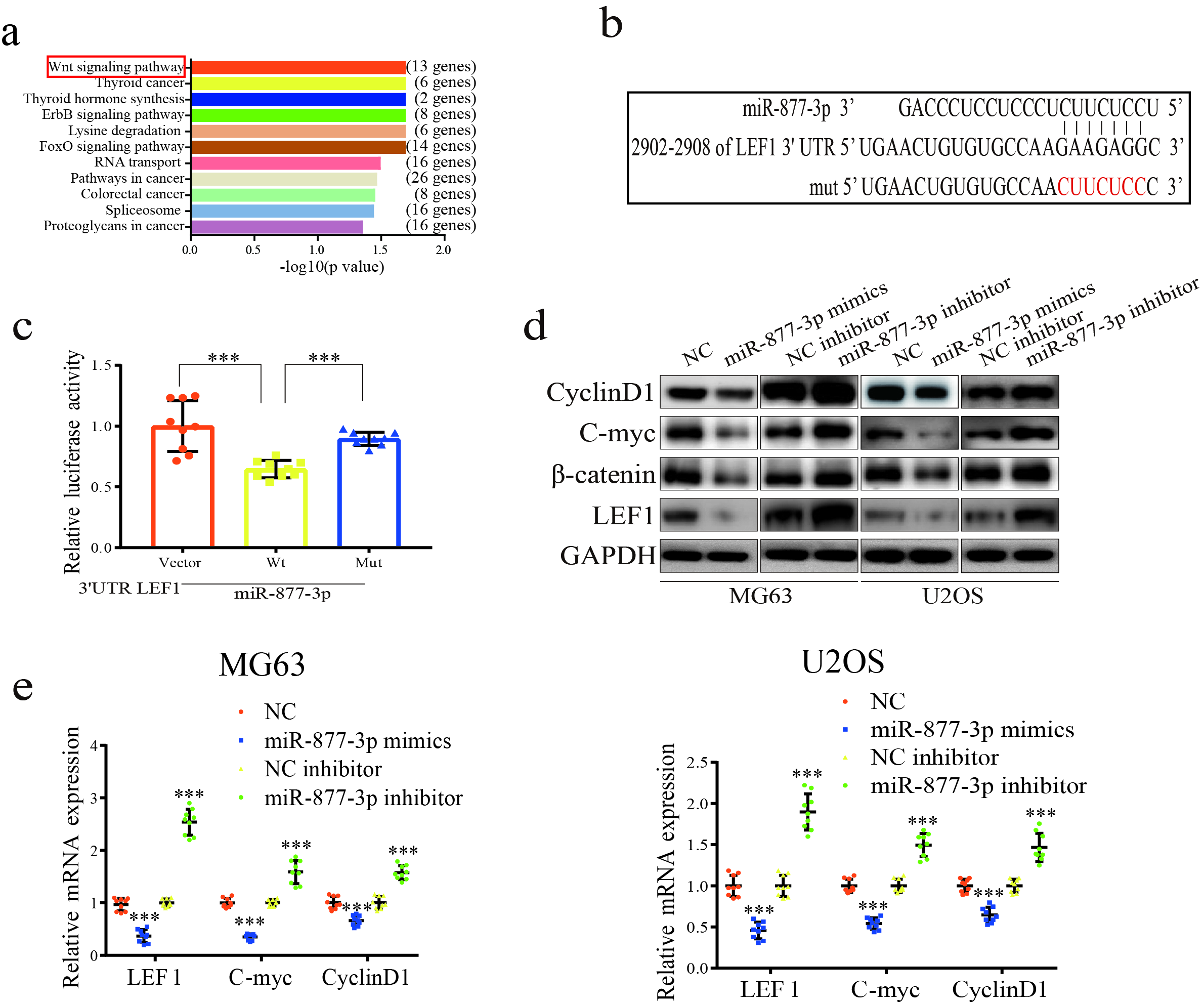

Supplement: Supplementary file 11 — Additional file 11: Figure S10. LEF1 is a direct target of miR-877-3p and miR-877-3p inhibits Wnt/β-catenin signaling. (a) Pathway enrichment analysis was conducted for miR-877-3p via mirPath v. 3. (b) Complementary sequences of the LEF1 3′ UTR for miR-877-3p. (c) 293 T cells were co-transfected with miR-877-3p and a luciferase reporter plasmid containing either wild type or mutated LEF1 3′ UTRs. Data represents the mean ± SD (n = 9). (d) The changes in levels of LEF1, β-catenin, C-myc, and CyclinD1 were detected by western blot. (e) Relative mRNA expression of LEF1, β-catenin, C-myc, and cyclinD1 was measured by qRT-PCR. Data represents the mean ± SD (n = 9). Three independent assays were performed in the above assays. (c, e) * P < 0.05, ** P < 0.01, *** P < 0.001 (Student’s t-test). [file 12943_2019_1076_MOESM11_ESM.tif]

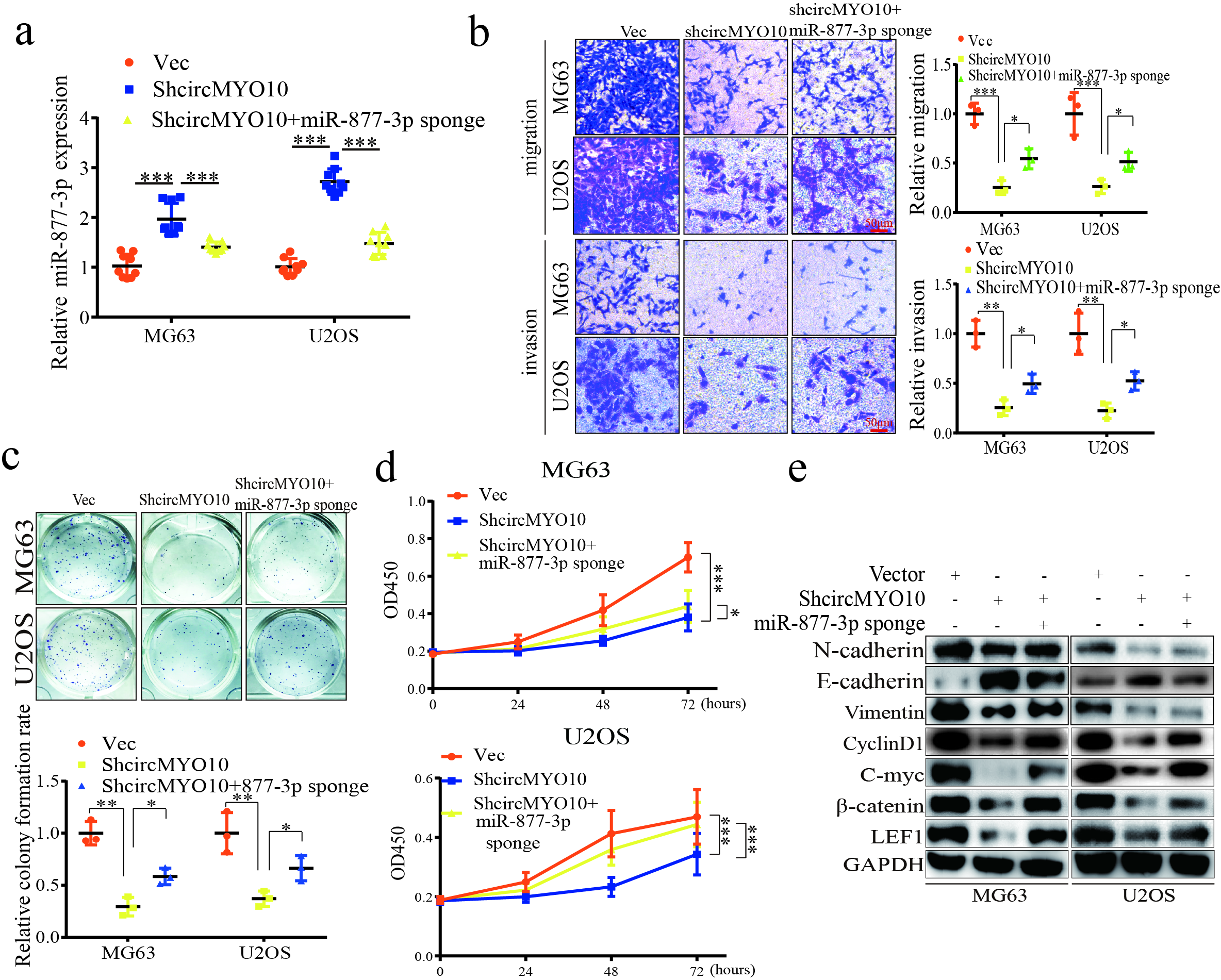

Supplement: Supplementary file 12 — Additional file 12: Figure S11. MiR-877-3p is involved in the process where circMYO10 promotes osteosarcoma progression via activated Wnt/β-catenin signaling. (a) The expression of miR-877-3p in MG63 cells and U2OS cells stably transfected with shcircMYO10 or shcircMYO10 plus miR-877-3p sponge. Data represents the mean ± SD. (b) Migration and invasion assay in transwells showed that miR-877-3p inhibition promoted the migration and invasion ability of cells stably transfected with shcircMYO10 and miR-877-3p overexpression induced contrary results. Scale bars = 50 μm. Data represents the mean ± SD. (c-d) Inhibition of miR-877-3p partially abrogated the suppressive effect of shcircMYO10 on proliferation ability of both MG63 and U2OS cells. Data represent the mean ± SD. (e) The effect of miR-877-3p on EMT and Wnt/β-catenin signaling in cells stably transfected with shcircMYO10 was measured by western blot. Three independent assays were performed in the above assays. (a-d) * P < 0.05, ** P < 0.01, *** P < 0.001 (Student’s t-test). [file 12943_2019_1076_MOESM12_ESM.tif]

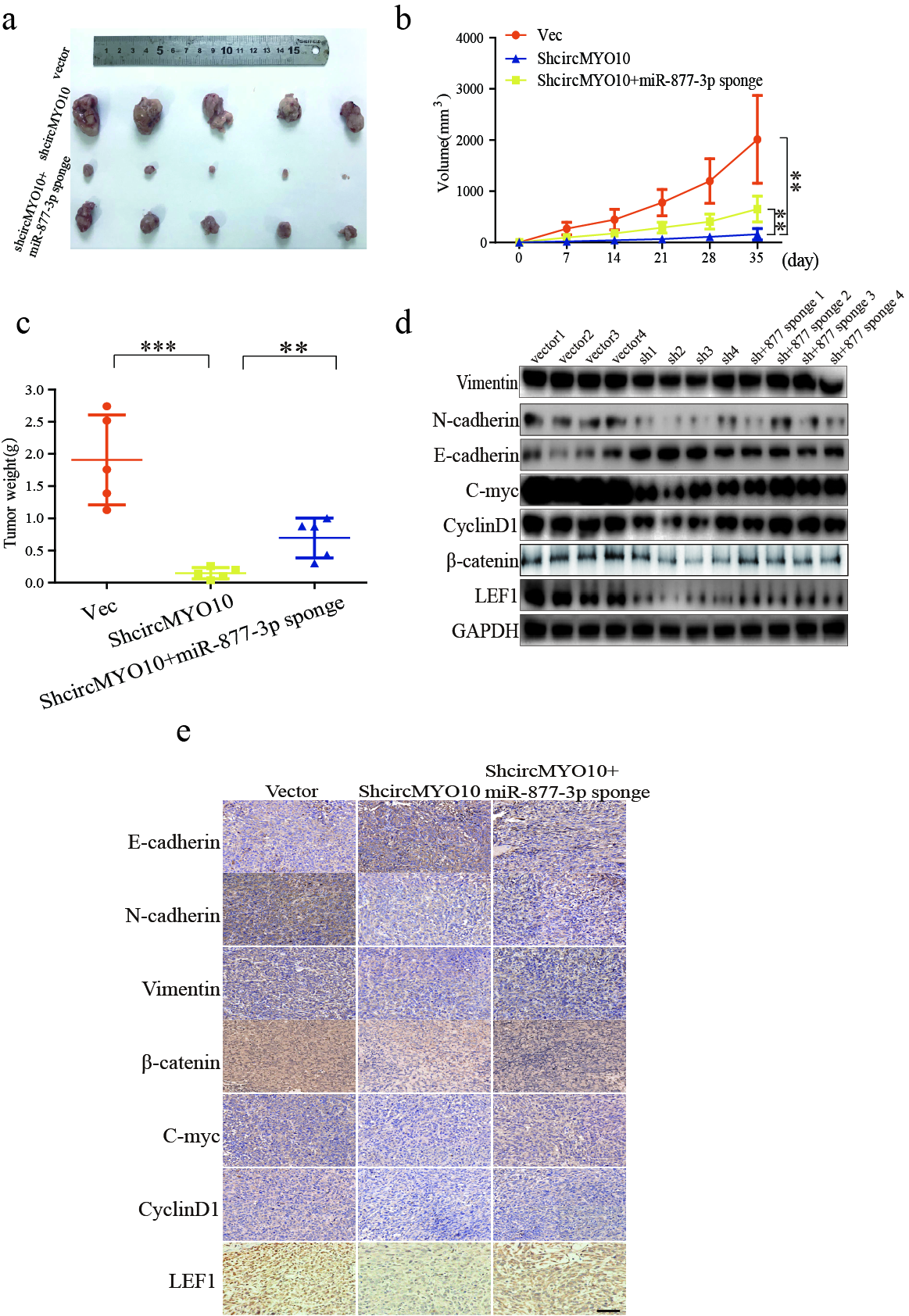

Supplement: Supplementary file 13 — Additional file 13: Figure S12. (a-c) Nude mice were injected subcutaneously with 107 stable MG63 control cells, cells transfected with shcircMYO10, or cells co-transfected with circMYO10 shRNA and miR-877-3p sponge. Tumors were dissected, photographed, and weighed after 5 weeks. (b) Tumor volume was measured every week. Data represent the mean ± SD (n = 5). (c) Average wet tumor weight in each group at the end of the experiment (day 35). Data represents the mean ± SD (n = 5). (d) Western blot analysis of LEF1, β-catenin, C-myc, CyclinD1, Vimentin, E-cadherin, and N-cadherin of protein extracted from tumors. Sh: shcircMYO10. Sh + sponge: shcircMYO10 + miR-877-3p sponge. (e) Immunohistochemistry analysis of LEF1, β-catenin, C-myc, CyclinD1, Vimentin, N-cadherin, and E-cadherin in tumors. Scale bars = 100 μm. Three independent assays were performed in the above assays. (c-d) * P < 0.05, ** P < 0.01, *** P < 0.001 (Student’s t-test). [file 12943_2019_1076_MOESM13_ESM.tif]
